# Topology‐Encoded Charge Polarity Governs Multiphase Organization in Intrinsically Disordered Protein Polymer Condensates

**DOI:** 10.1002/smll.74740

**Published:** 2026-07-28

**Authors:** Julio Fernández‐Fernández, Vicente Domínguez‐Arca, Raúl Escribano, Sheila Lorenzo, Sergio Ferrero, Sergio Acosta, José Carlos Rodríguez‐Cabello

**Affiliations:** ^1^ Bioforge Laboratory (Group for Advanced Materials and Nanobiotechnology) Laboratory for Disruptive Interdisciplinary Science (LaDIS) CIBER‐BBN, Edificio LUCIA Universidad De Valladolid Valladolid Spain; ^2^ Biosystems and Bioprocess Engineering (Bio2Eng) Group Institute of Marine Research of the Spanish Research Council IIM‐CSIC Vigo Spain; ^3^ Colloids Physical and Biophysical Chemistry Bielefeld University Bielefeld Germany; ^4^ GIR MIOMeT, IU CINQUIMA/Química Inorgánica Facultad De Ciencias Universidad De Valladolid Valladolid Spain

**Keywords:** electrostatic complementarity, IDPPs, multiphase compartmentalization, synthetic condensates

## Abstract

Synthetic biomolecular condensates offer a route to engineer compartmentalized microenvironments with tunable physicochemical properties, yet design principles for controlling their internal organization remain limited. Here, we report a minimal two‐component system based on thermoresponsive intrinsically disordered protein polymers with lower critical solution temperature behavior (LCST‐IDPPs), in which electrostatic topology programs liquid–liquid phase separation (LLPS). Incorporating charged residues into LCST‐IDPPs suppresses phase separation under physiological conditions, whereas mixing oppositely charged, individually non‐coacervating IDPPs restores LLPS as an emergent, composition‐dependent process driven by multivalent intermolecular charge compensation. We compare this two‐component system with a covalently linked diblock containing the same charged chains. This change in chain connectivity alters the coupling between electrostatic pairing, counterion redistribution, and LCST‐driven dehydration. As a result, the balance between inter‐ and intrachain ionic pairing encodes the residual charge and micropolarity of the dense phase. This topology‐dependent microenvironment controls condensate miscibility and drives the formation of either homogeneous or internally demixed multiphase assemblies. The condensate interior also shifts the apparent pK_a_ of ionizable residues, indicating that phase separation modifies local acid–base equilibria and alters the effective side‐chain charge. Together, these findings show how electrostatic topology influences LLPS, the dense‐phase microenvironment, and mesoscale organization in IDPP condensates.

## Graphical abstract

Electrostatic topology is shown to program phase separation and internal organization in thermoresponsive intrinsically disordered protein polymer (IDPP) condensates. By comparing oppositely charged IDPPs with a covalent diblock, chain connectivity is revealed to control charge compensation, dense‐phase micropolarity, miscibility, and apparent acid‐base equilibria, providing a minimal framework for designing multiphase synthetic condensates.

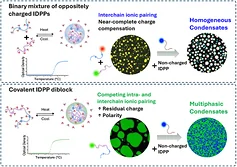

## 1 Introduction

Supramolecular assemblies generated from short peptides and protein polymers have become a powerful and chemically minimalist platform for bottom‐up biofabrication of functional biomaterials and bioactive interfaces [1, 2]. Many established systems rely on ordered secondary‐structure motifs such as β‐sheets, coiled coils, or α‐helices that encode tunable architectures such as fibrils, nanorribons, protein cages, giant vesicles, liquid droplets or shear‑thinning hydrogels [3, 4, 5, 6, 7, 8]. While these „ordered″ assemblies offer structural predictability and mechanical robustness, their stability can limit reversibility and dynamic responsiveness.

Intrinsically disordered proteins (IDPs) and their synthetic analogues, intrinsically disordered protein polymers (IDPPs) provide an alternative design paradigm based on dynamic, multivalent interactions rather than fixed secondary structure [9, 10]. Through liquid‐liquid phase separation (LLPS) of IDPs, cells can form biomolecular condensates to compartmentalize biochemical processes without a surrounding membrane [11]. These condensates selectively enrich or exclude ions, small molecules, RNAs, and proteins, thereby regulating buffer fluctuations [12, 13], or coordinating biochemical reactions and signaling pathways [14]. These observations highlight that structural disorder is not a liability, but a programmable feature that couples sequence‐encoded interactions to mesoscale organization and function.

Building upon this biological paradigm, IDPPs have been developed as recombinant building blocks that not only recapitulate key features of native IDPs but also extend their programmability [15, 16]. Their recombinant nature enables precise control over chain length, sequence composition, and molecular architecture. The molecular grammar governing IDPP condensation is increasingly understood [17, 18, 19], and can be broadly divided into two regimes [16]. Resilin‐like sequences enriched in aromatics and charged residues rely on *π*–*π*, cation–π, electrostatic, and hydrophobic interactions, and often exhibit upper critical solution temperature (UCST) phase behavior. By contrast, elastin‐like repeats such as VPGXG primarily undergo entropy‐driven hydrophobic collapse, leading to lower critical solution temperature (LCST)‐mediated coacervation. These two regimes thus represent the enthalpy‐ and entropy‐dominated extremes of protein phase separation, respectively. While these general principles for LLPS are established, design rules that control not only phase boundaries but also dense‐phase polarity, residual charge, and internal organization via sequence architecture remain limited.

Synthetic condensates created from IDPPs are increasingly being explored as functional modules that mimic cellular organization. Such assemblies can create catalytic microenvironments [20], modulate ion partitioning and electrochemical potentials at condensate interfaces [13, 21, 22], and serve as programmable compartments for sequestration and release of biomolecules [23, 24]. Moreover, tuning the amino‐acid composition of IDPPs allows control of condensate properties [18, 19], including phase behavior [25], viscoelasticity [26], and micropolarity [27], a recently identified determinant of multilayered condensate organization.

Beyond single‐component systems, biological LLPS almost always arises from both homotypic and heterotypic interactions [28, 29]. In living cells, condensate stability and composition are not fixed properties of isolated biomolecules, but emerge from the thermodynamics of multicomponent mixtures. Heterotypic interactions between distinct biomolecules typically provide the cohesive forces that stabilize condensates, whereas homotypic associations modulate their selectivity and internal organization [28]. Translating this principle to synthetic systems introduces an additional layer of design complexity. In particular, the balance between homotypic associations within each protein and heterotypic interaction between distinct partners can modulate condensate miscibility, internal organization, and responsiveness, as shown in both short peptide and IDPP‐based condensates [27, 30, 31]. Understanding how this competition shapes phase behavior is therefore critical for the rational design of bicomponent IDPP condensates with controlled physicochemical properties.

While previous studies have examined peptide and protein‐based condensates [31, 32, 33, 34, 35], the role of electrostatic topology in programming phase behavior and dense‐phase microenvironment within a minimal, LCST‐IDPP pair remains unclear. Establishing design rules for such assemblies is both a mechanistic necessity and a transformative opportunity. Here, we develop a minimal, two‐component LCST‐IDPP platform to elucidate how electrostatic complementarity and molecular architecture influence condensate formation. By comparing sequence‐matched, oppositely charged IDPPs and a covalently linked diblock analogue, we decouple inter‐ and intramolecular ionic pairing while maintaining constant overall composition. Using calorimetry, confocal microscopy, turbidity assays, circular dichroism, nuclear magnetic resonance (NMR), and atomistic simulations, we demonstrate that chain architecture controls salt‐bridge formation, thereby modulating dehydration cooperativity and the apparent residual charge within the dense phase. Furthermore, we demonstrate that the resulting residual charge encodes condensate micropolarity and drives either homogeneous or compartmentalized organization upon coacervation with hydrophobic IDPPs. Together, these results identify charge topology as a molecular design parameter for tuning phase behavior, internal chemistry and multiphase organization in synthetic protein condensates.

## 2 Results and Discussion

### 2.1 Coacervation in Solution

**FIGURE 1:**
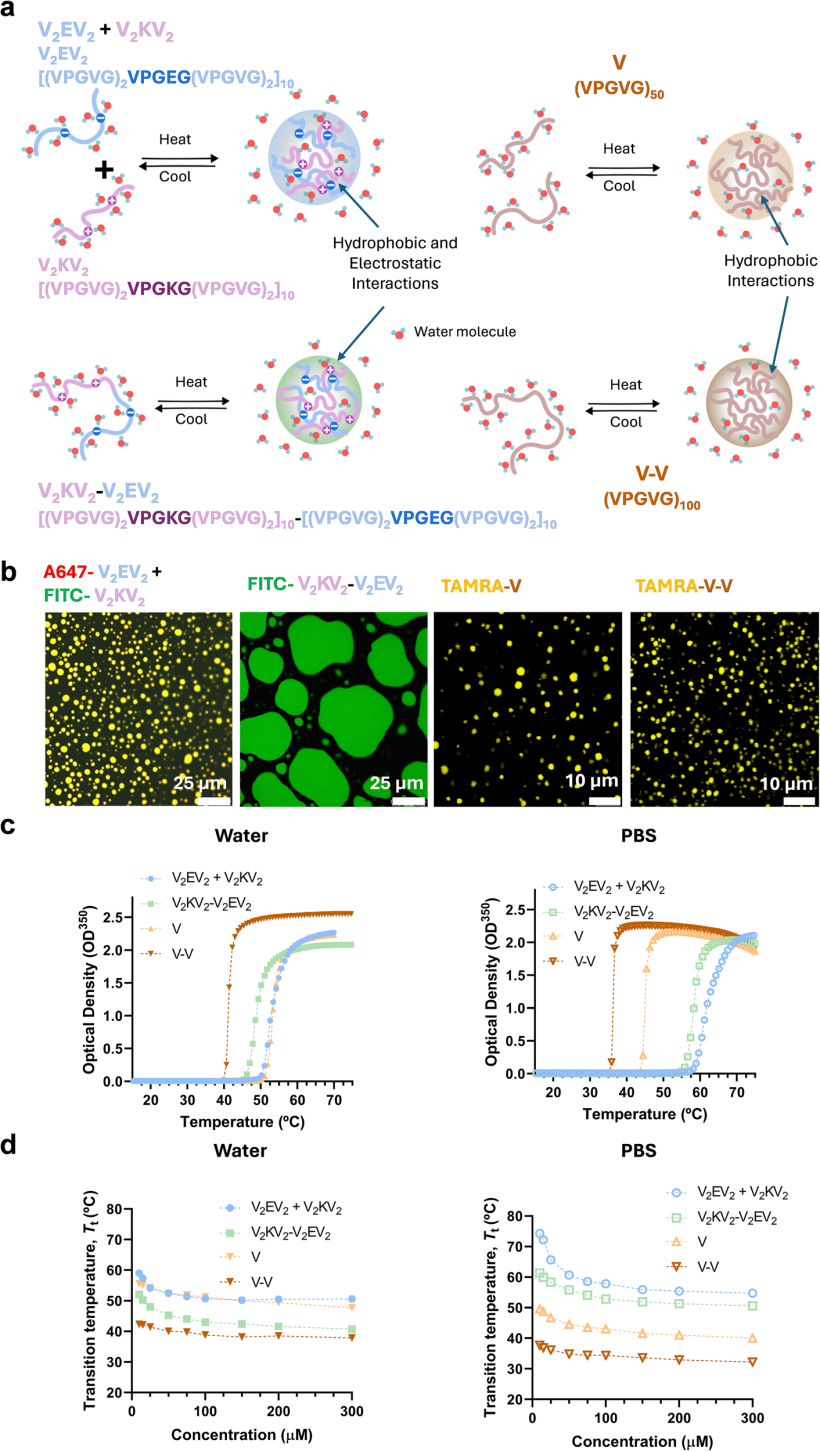
Sequence architecture dictates LLPS and condensate morphology. (a) Schematic representation of five IDPPs and their LLPS in aqueous solution: the equimolar mixture (V_2_EV_2_ + V_2_KV_2_) of two oppositely charged IDPPs (i.e., the cationic V_2_KV_2_ and the anionic V_2_EV_2_), the corresponding covalently linked diblock copolymer (V_2_KV_2_‐V_2_EV_2_), and two neutral variants, (VPGVG)_50_ (V) and (VPGVG)_100_ (V‐V). Charge complementarity in V_2_KV_2_/V_2_EV_2_ systems enables both electrostatic and hydrophobic interactions during condensation, whereas VPGVG‐based homopolymers undergo phase separation solely through hydrophobic collapse. (b) Confocal micrographs of the condensates formed with fluorescently labelled IDPPs in type I water at 250 µm. After thermally triggered LLPS, all IDPPs formed spherical condensates that remained in solution except V_2_KV_2_‐V_2_EV_2_, which displayed more extensive coalescence, giving rise to larger condensates that sedimented. Images of V_2_EV_2_ + V_2_KV_2_, V, and V‐V were acquired 25–35 µm above the glass surface, while V_2_KV_2_‐V_2_EV_2_ was imaged directly at the glass surface. Scale bars: 25 µm (V_2_EV_2_ + V_2_KV_2_ and V_2_KV_2_‐V_2_EV_2_, left), 10 µm (V and V‐V, right). (c) UV/Vis turbidimetry profiles and d) phase diagrams in water and PBS reveal the impact of ionic strength in modulating LLPS. In charged systems (V_2_EV_2_ + V_2_KV_2_ and V_2_KV_2_‐V_2_EV_2_), electrostatic screening increases the cloud point temperature (*T*_t_), consistent with reduced effective attraction upon charge neutralization. In contrast, in hydrophobic IDPPs (V and V‐V), salt lowers *T*_t_, indicating enhanced hydrophobic dehydration and chain collapse. IDPP concentrations in (c) were normalized to the number of pentapeptide repeats: 25 µm for V‐V and V_2_KV_2_‐V_2_EV_2_, and 50 µm for V_2_EV_2_ + V_2_KV_2_ and V.

To determine the role of electrostatic topology in LCST‐driven phase behavior, we engineered a library of elastin‐like IDPPs based on the canonical elastin‐like pentapeptide motif, VPGVG (Figure 1a, Tables S1 and S2, Figures S1 and S2). In these constructs, one in every five pentapeptides was substituted at the guest (X) position with either glutamic acid (Glu, E) or lysine (Lys, K), generating two oppositely charged 50‐mer polypeptides, designated V_2_EV_2_ and V_2_KV_2_, respectively. This design preserved the hydrophobic backbone responsible for LCST behavior while introducing a controlled density of ionizable residues, thereby encoding competing electrostatic interactions: repulsive homotypic contacts between like‐charged chains and attractive heterotypic interactions between oppositely charged partners. To isolate the role of molecular architecture, we also constructed a covalently linked diblock copolymer (V_2_KV_2_‐V_2_EV_2_) and two non‐charged reference polymers, (VPGVG)_50_ (V) and (VPGVG)_100_ (V‐V).

Individually, and as expected, V_2_EV_2_ and V_2_KV_2_ showed no measurable cloud point (Figure S3), consistent with electrostatic repulsion and increased chain polarity that broadens their solubility window across temperatures [36]. In contrast, the equimolar mixture (V_2_EV_2_ + V_2_KV_2_) exhibited a clear and reversible phase separation (Figure 1c and Figure S4), producing IDPP‐rich microdroplets above a well‐defined cloud point transition temperature (*T*_t_), close to that of the uncharged analogue V (Figure 1c,d). Quantitative colocalization analysis revealed strong spatial correlation between both components, with Pearson's R = 0.98 and R = 0.89 after Costes automatic thresholding, together with thresholded Manders coefficients of tM1 = 0.751 and tM2 = 0.679, supporting substantial spatial overlap of both IDPPs within the condensate‐rich phase.

The diblock IDPP, V_2_KV_2_‐V_2_EV_2_, also underwent a well‐defined LCST‐type LLPS, but displayed distinct condensate growth and interfacial behavior. Immediately above *T*_t_, IDPP‐rich domains appeared in solution, consistent with the spinodal demixing behavior reported for elastin‐like polypeptides (ELPs) [31]. In contrast to the V_2_EV_2_ + V_2_KV_2_ mixture, V_2_KV_2_‐V_2_EV_2_ condensates coalesced into larger condensates that progressively settled onto the bottom glass surface (Figure S5). This surface interaction was particularly evident on Pluronic F127‐treated glass, where the condensates showed pronounced wetting and irregular spreading (Figure 1b), whereas pristine glass favored more rounded condensates (Figure S6). This behavior differs from that reported for other IDP condensates, such as LAF‐1 [37], and may reflect the amphiphilic character of the diblock architecture, which could promote adsorption and wetting at the aqueous‐solid interface.

To compare the bulk behavior with phase separation under microconfinement, we also monitored temperature‐induced condensation after encapsulating the IDPP solutions in water‐in‐oil microdroplets (Figure S7). Previous studies on ELPs confined in microdroplets have shown that phase separation proceeds through rapid and spatially distributed formation of protein‐rich mesoscopic domains, consistent with spinodal decomposition, followed by coalescence and coarsening [31]. In agreement with this framework, all IDPP systems displayed thermally triggered formation of IDPP‐rich domains inside the microdroplets. However, their subsequent coarsening depended strongly on IDPP architecture. The charged systems evolved toward one dominant condensate that concentrated most of the IDPP within each compartment. By contrast, and similar to bulk solution, the VPGVG‐based constructs, V and V‐V, retained multiple IDPP‐rich domains within the same microdroplet over the observation window, indicating less complete coalescence under confinement. This lower degree of coarsening may be partly related to the experimental conditions, including the use of type I water and the presence of the short N‐terminal leader sequence (Table S1). Although V and V‐V are designed as non‐charged hydrophobic controls, this leader contains one Glu residue and may introduce weak charge and hydration contributions that modulate condensate mobility and coalescence, particularly under low‐ionic‐strength conditions. Within this shared behavior, V‐V showed a higher density of IDPP‐rich domains than V, consistent with its longer VPGVG chain and higher hydrophobic valency.

Under physiological pH and ionic strength, i.e., in PBS (ionic strength 0.156 M, pH 7.4), the systems exhibit the expected interplay between charge screening and salting‐out. Charged systems showed an increase on *T*_t_ values compared to those found in water, whereas the non‐charged IDPPs displayed the opposite trend, with salt lowering *T*_t_, due to the well‐known salting‐out effect that promotes hydrophobic dehydration and chain collapse [38]. Notably, although V‐V and V_2_KV_2_‐V_2_EV_2_ contain the same number of pentapeptide repeats, their chain‐length effects on *T*_t_ were not equivalent. In both cases, increasing molecular weight lowered the *T*_t_ relative to their shorter analogues, consistent with the cooperative dehydration typical of LCST IDPPs, whereby longer chains promote more extensive desolvation and aggregation [39]. However, this effect was markedly attenuated in the charged diblock, suggesting that the electrostatic intrachain interactions partially disrupt cooperative desolvation.

### 2.2 Two Energy Landscapes Revealed by Ionic Strength and Molecular Architecture

**FIGURE 2:**
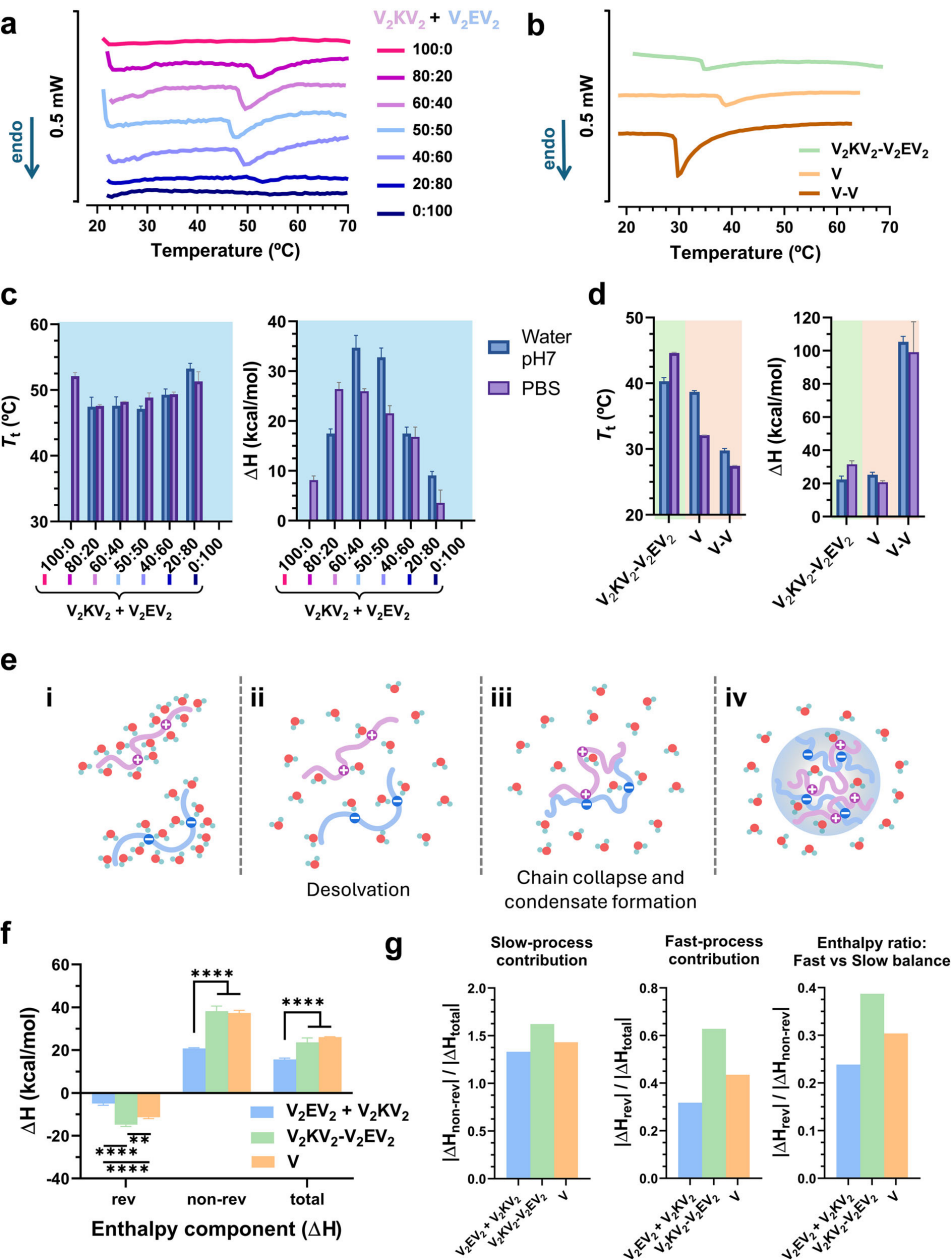
Thermodynamic evaluation reveals the influence of charge stoichiometry, IDPP architecture and hydrophobicity on the LLPS. (a,b) Conventional DSC thermograms of (a) mixtures of V_2_KV_2_ and V_2_EV_2_ at different molar ratios (100:0 to 0:100), and (b) of the diblock copolymer V_2_KV_2_‐ V_2_EV_2_ and the hydrophobic IDPPs V and V‐V reveal distinct endothermic profiles depending on the molecular architecture. (c,d) Transition temperatures (*T*_t_) and total enthalpy changes (ΔH_tot_) extracted from conventional DSC data plotted for (c) mixtures of V_2_KV_2_ + V_2_EV_2_ at varying charge ratios and (d) the V_2_KV_2_‐ V_2_EV_2_, V, V‐V systems. The binary V_2_KV_2_ + V_2_EV_2_ system shows an asymmetric composition dependence, with the highest ΔH values observed in the equimolar to slightly V_2_KV_2_‐rich mixtures. (e) Schematic representation of the proposed LCST‐driven condensation mechanism. (i) Below the *T*_t_, the IDPP chains are hydrophobically hydrated. (ii) Upon heating above *T*_t_ (LCST), IDPP chains undergo cooperative desolvation, followed by (iii) chain collapse and intra‐ and intermolecular interactions leading to (iv) condensate formation and coalescence. (f) Deconvolution of enthalpy components via TM‐DSC. Reversing component (ΔH_rev_) corresponds to fast, cooperative, and predominantly endothermic processes, specifically the formation of hydrophobic and electrostatic interactions that drive phase separation. The non‐reversing component (ΔH_non‐rev_) arises from slower processes, such as water diffusion and release which are physically reversible but occurs on longer timescales. (g) Enthalpic ratios derived from TM‐DSC deconvolution highlight the relative weight of fast and slow processes in LLPS. Left: ΔH_non‐rev_/ΔH_total_ represents the contribution of slow processes. Center: ΔH_rev_/ΔH_total_ reports the contribution of fast processes, i.e., the fraction of the transition enthalpy compensated by rapid reversing events. Right: ΔH_rev_/ΔH_non‐rev_ indicates the ratio between both components.

To quantify how charge stoichiometry and molecular architecture shape the energetics of LLPS, we analyzed the thermal transitions of V_2_EV_2_/V_2_KV_2_ mixtures at varying stoichiometries using differential scanning calorimetry (DSC) (Figure 2a,c and Figure S8a). The observed endotherms report on the energetic cost of disrupting polypeptide‐water hydrogen bonds and releasing hydration layers and counterions, compensated by favorable polypeptide‐polypeptide interactions in the polypeptide‐rich phase. Accordingly, higher transition enthalpies (ΔH) reflect more extensive cooperative reorganization during LLPS.

In water (pH 7), neither V_2_EV_2_ nor V_2_KV_2_ alone underwent LLPS, whereas all mixtures of V_2_EV_2_ and V_2_KV_2_ underwent endothermic LLPS with ΔH in the range of 9–35 kcal mol^−1^, depending on the stoichiometry. The highest ΔH values were observed around the equimolar and slightly V_2_KV_2_‐rich compositions, (ΔH_60:40_ = 34.7 ± 2.5 kcal·mol^−1^and ΔH_50:50_ = 32.8 ± 1.9 kcal mol^−1^). This asymmetric maximum suggests that Lys and Glu‐ containing chains do not contribute equally to the coupled processes of charge compensation, hydration, counterion redistribution, and multivalent network formation. Similar deviations from strict equimolarity have been reported in polyelectrolyte complexes and charge‐complementary peptide systems, where cationic excess can modulate coassembly, and higher‐order organization [40, 41].

Increasing ionic strength markedly reshaped this energetic landscape. In PBS, the enthalpies of V_2_EV_2_/V_2_KV_2_ mixtures varied in comparison to water, highlighting the influence of salts on the balance between electrostatic screening, side‐chain hydration, counterion association, and dehydration of the elastin‐like backbone (Figure 2c and Figure S8a). V_2_KV_2_‐rich mixtures (80:20 and 60:40) showed the highest ΔH values (e.g., ΔH_80:20_ = 26.4 ± 1.4 kcal·mol^−1^ and ΔH_60:40_ = 26.0 ± 0.5 kcal·mol^−1^), whereas V_2_EV_2_‐rich systems exhibited strongly reduced values (ΔH_40:60_ = 16.8 ± 2.0; ΔH_20:80_ = 3.6 ± 2.6 kcal·mol^−1^). Remarkably, V_2_KV_2_ alone (100:0), which remained fully soluble in water, underwent LLPS in PBS (ΔH_100:0_ = 8.15 ± 0.83 kcal·mol^−1^).

This behavior suggests that the phase separation of V_2_KV_2_ in PBS is not governed by Debye screening alone, but by a combination of electrostatic screening, counterion association, and ion‐specific dehydration. Screening of Lys–Lys repulsion can lower the energetic penalty for homotypic V_2_KV_2_ association, while chloride and phosphate anions may further promote condensation by associating with Lys‐NH_3_^+^ groups and reducing side‐chain hydration. In parallel, salts such as NaCl and phosphate can favor dehydration of the elastin‐like backbone, thereby lowering the apparent LCST of ELP‐based systems [27, 33]. The different response of V_2_KV_2_ and V_2_EV_2_ supports this interpretation: whereas Lys‐NH_3_^+^ groups are less favorably solvated than Glu carboxylates [42], the persistent solubility of V_2_EV_2_, even at high concentration (5% w/v), is consistent with the robust hydration of carboxylate groups [43].

Molecular architecture modulated these trends. The covalently‐bonded diblock (V_2_KV_2_‐V_2_EV_2_) underwent LLPS at lower *T*_t_ and ΔH (40.3°C; 22.4 ± 2.1 kcal·mol^−1^, respectively) than the equimolar mixture (V_2_EV_2_ + V_2_KV_2_) (47.1°C; 32.8 ± 1.9 kcal·mol^−1^) in water, consistent with intrachain charge pairing that may partially frustrate cooperative dehydration. In PBS, however, the behavior inverted, and V_2_KV_2_‐V_2_EV_2_ exhibited a significantly higher ΔH (31.6 ± 2.1 kcal·mol^−1^) and a stronger shift in *T*_t_ (44.6°C) compared to the equimolar mixture. This inversion suggests that under physiological ionic strength, charge screening may impair intermolecular electrostatic attraction in the two‐component system, but intramolecular salt bridges within the V_2_KV_2_‐V_2_EV_2_ system are less affected by bulk counterions and the salting‐out effect is enhanced at high concentrations.

As expected, non‐charged IDPPs (V and V‐V) followed canonical LCST behavior. In water, V exhibited a lower *T*_t_ than the V_2_EV_2_/V_2_KV_2_ mixture, with a ΔH of 25.3 ± 1.5 kcal·mol^−1^. Doubling the chain length to V‐V further decreased *T*_t_ and produced a non‐linear increase in enthalpy to 105.4 ± 3.2 kcal·mol^−1^, consistent with previous studies on VPGVG polymers [44]. In PBS, both underwent salting‐out, lowering their *T*_t_ relative to water. Their enthalpies again differed with chain length, consistent with stronger cooperative dehydration at higher molecular weights.

### 2.3 Thermodynamic Dissection of LCST‐Type Phase Separation

LCST phase separation in IDPP condensates involves thermodynamically coupled processes that occur on different timescales (schematically represented in Figure 2e) [44, 45]. The first contribution is a cooperative, enthalpically costly solvent‐reorganization process associated with the expulsion of structured hydration water during hydrophobic desolvation and counterion redistribution. In elastin‐like systems, heating above *T*_t_ promotes rapid dehydration of the elastin‐like backbone and hydrophobic association, producing an endothermic but entropy‐favored phase separation. However, the reverse process is not necessarily equally fast. Rehydration and redissolution require water to repartition into the polymer‐rich phase, disrupt polymer‐polymer contacts, restore the hydration shells, and redistribute counterions between the dense and dilute phases. The second contribution arises from faster local rearrangements within the emerging dense phase, including chain reorganization and, in charged LCST‐IDPPs, transient electrostatic pairing. Thus, the measured transition enthalpy reflects the balance between the endothermic cost of solvent release and phase separation, and exothermic contributions associated with local molecular reorganization within the dense phase.

To experimentally resolve these coupled processes, we employed temperature‐modulated DSC (TM‐DSC) to decompose the total transition enthalpy (ΔH_tot_) into its non‐reversing (slow) and reversing (fast) components (Figure 2f,g). We interpret the non‐reversing term (ΔH_non‐rev_) as a measure of heat‐flow contributions that do not fully equilibrate on the timescale of the temperature modulation. These contributions are associated primarily with cooperative hydrophobic hydration and, where applicable, counterion redistribution. By contrast, the reversing component (ΔH_rev_) reflects faster heat‐flow contributions that can follow the imposed modulation, including local chain rearrangements and, where oppositely charged residues are present, salt‐bridge formation within the dense phase.

Across all systems, ΔH_non‐rev_ dominated the transition, yielding positive ΔH_tot_ values consistent with conventional DSC. Quantitatively, the non‐charged IDPP, V, showed a large slow component (ΔH_non‐rev_ = 37.4 ± 1.2 kcal·mol^−1^) partially compensated by a modest fast exotherm (ΔH_rev_ = ‐11.4 ± 0.6 kcal·mol^−1^), resulting in a ΔH_total_ = 26.1 ± 0.2 kcal·mol^−1^. V_2_KV_2_‐V_2_EV_2_ displayed a similar slow term (ΔH_non‐rev_ = 38.2 ± 2.4 kcal·mol^−1^), but a larger reversing contribution (ΔH_rev_ = ‐14.8 ± 0.8 kcal·mol^−1^), reducing ΔH_total_ to 23.6 ± 2.1 kcal·mol^−1^. In contrast, the equimolar V_2_EV_2_ + V_2_KV_2_ mixture showed the smallest enthalpies, with ΔH_non‐rev_ = 20.7 ± 0.4 kcal·mol^−1^ and ΔH_rev_ = ‐4.9 ± 0.8 kcal·mol^−1^ (ΔH_total_ = 15.6 ± 0.7 kcal·mol^−1^).

These differences reveal how molecular architecture and salt‐bridge topology modulate not only the total enthalpy of LLPS, but also the relative contributions of slow and fast enthalpic components. Uncharged IDPPs (V) undergo LLPS primarily through slow, cooperative dehydration and hydrophobic association. The diblock V_2_KV_2_‐V_2_EV_2_ exhibits a similar slow enthalpic cost, but a larger fast compensatory contribution. This behavior is consistent with the high local concentration of oppositely charged segments linked covalently, which may favor rapid local electrostatic rearrangements and intrachain or short‐range interchain charge pairing prior to macroscopic phase separation. In this architecture, charge compensation and hydrophobic collapse are topologically coupled within the same polypeptide chain, allowing multivalent contacts to reorganize locally during dehydration.

By contrast, in the V_2_EV_2_ + V_2_KV_2_ mixture, charge neutralization depends on heterotypic interactions among many separate chains within a two‐component condensate network. Thus, Lys‐Glu contacts must be formed intermolecularly, while competing with like‐charge repulsion, chain entropy, and hydration of the charged segments. As a result, dehydration proceeds in a less cooperative and energetically weaker manner, reflected in the lower ΔH_non‐rev_ and ΔH_rev_ values.

Analysis of enthalpy ratios further emphasizes these differences (Figure 2g). Although slow processes dominate the transition in all systems (ΔH_non‐rev_/ΔH_total_ > 1), the relative fast contribution is highest for V_2_KV_2_‐V_2_EV_2_ (∼63%), intermediate for V (∼43%), and minimal for the binary mixture (∼32%). This trend supports the conclusion that stoichiometry and molecular architecture modulate not only the total enthalpy of LLPS, but also the balance between slow rehydration and faster local reorganization. The enhanced fast contribution in V_2_KV_2_‐V_2_EV_2_ is consistent with intrachain electrostatic reorganization and salt‐bridge formation, which provide rapid enthalpic compensation absent in the non‐charged system. By contrast, the attenuated fast contribution in the V_2_EV_2_ + V_2_KV_2_ mixture suggests that, when Lys‐Glu salt‐bridges are formed only interchain, these interactions become more distributed and less topologically constrained within the condensate network. This would explain why ΔH_total_ is lowest in the mixture despite the presence of both charged residues, while V and V_2_KV_2_‐ V_2_EV_2_ exhibit stronger cooperative reorganization that translates into higher ΔH_total_. Consistently, these enthalpic differences align with the distinct secondary structures observed across condensates (Figures S9 and S10).

Control experiments performed under strongly acid and basic conditions further clarified this picture (Figure S11). Below and above their respective pK_a_ values (i.e., pK_aE_ and pK_aK_), both V_2_EV_2_ and V_2_KV_2_ lost electrostatic charges and behaved as non‐charged IDPPs with clear endotherms. V_2_KV_2_ at pH 12 displayed ΔH_total_ = 27.7 ± 0.3 kcal·mol^−^^1^ (dominated by ΔH_non‐rev_ = 33.8 ± 1.1). V_2_EV_2_ at pH 3 showed an even larger enthalpic value (ΔH_total_ = 42.2 ± 0.1, ΔH_non‐rev_ = 49.6 ± 1.6), consistent with stronger cooperative dehydration of protonated carboxylic groups compared to Lys amine groups.

These findings also clarify how the present LCST‐IDPP condensates differ from LLPS‐mediated nucleation pathways [46, 47]. In those systems, metastable solute‐rich droplets can act as precursor states that lower the nucleation barrier toward more ordered structures. By contrast, phase separation in the present system is better described as spinodal‐like demixing, where IDPP‐rich domains emerge throughout the sample as local composition fluctuations are amplified under thermodynamically unstable conditions [31]. Thus, the salt‐bridge and hydrophobic‐contact‐enriched regions should not be interpreted as stable nuclei or glass‐forming precursors [48], but as transient local interaction motifs that contribute to heterogeneous organization within the dense phase while preserving the hydrated, reversible, and thermoresponsive character of the condensates.

### 2.4 A Two‐Component IDPP System Undergoes Asymmetric, Entropy‐Governed Condensation

To connect the calorimetric energy partition with molecular interactions, we combined isothermal titration calorimetry (ITC) with atomistic molecular dynamics (MD) simulations. ITC provides apparent binding stoichiometries, association constants, and enthalpic signatures associated with the interaction between V_2_EV_2_ and V_2_KV_2_ during condensate formation, while MD visualizes chain compaction, salt‐bridge formation, and counterion release across *T*_t_. Together, these measurements test whether the asymmetric enthalpy maximum and architecture‐dependent ΔH_rev_ contributions observed by DSC arise from unequal charge compensation and collective solvent reorganization at the molecular level.

**FIGURE 3:**
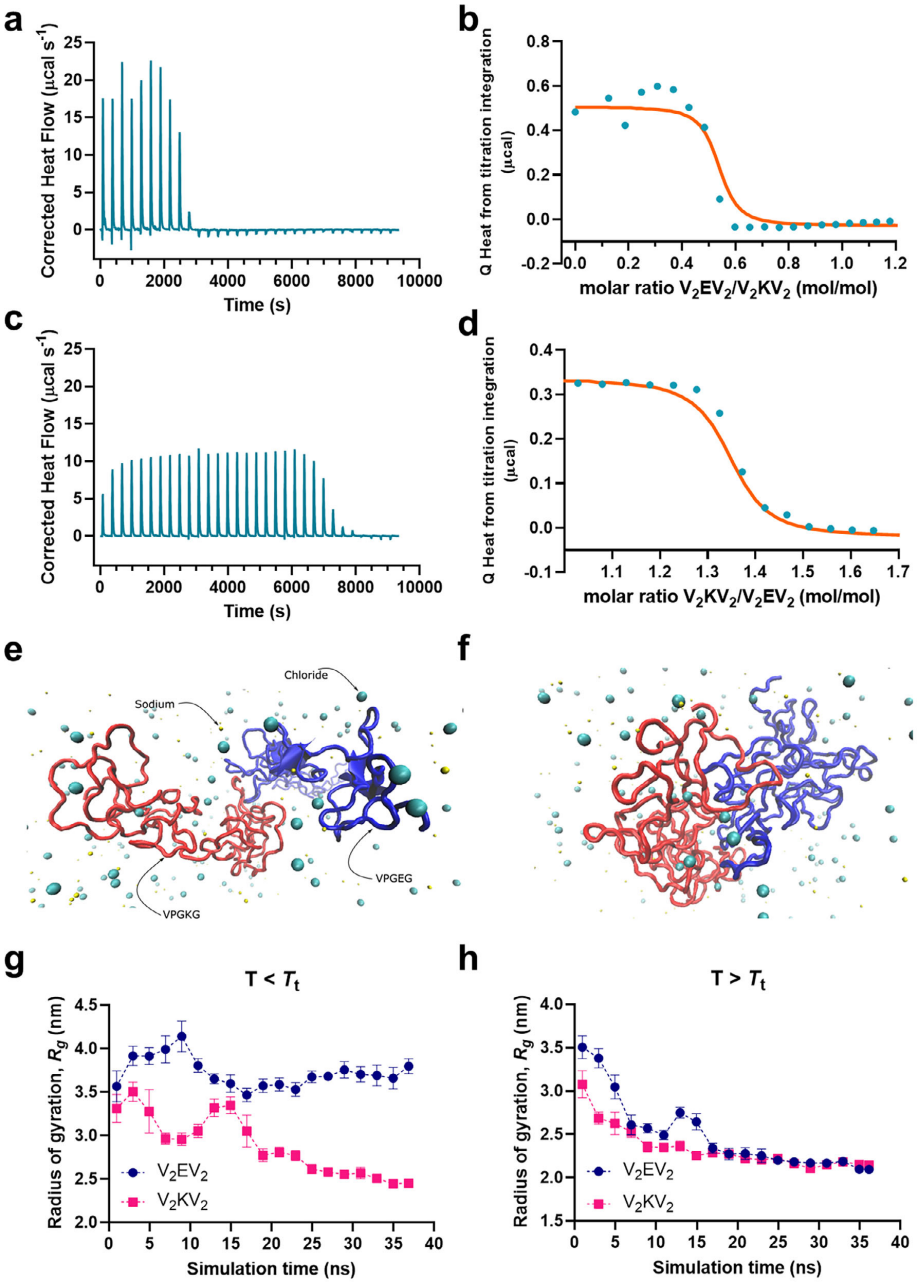
Thermoresponsive LLPS between V_2_EV_2_ and V_2_KV_2_ as revealed by ITC and molecular simulations. (a) Representative raw calorimetric data with baseline correction for V_2_EV_2_→V_2_KV_2_ titration above the *T*_t_ (T = 60°C). (b) Corresponding binding isotherm and nonlinear fit. (c) Raw calorimetric data for V_2_KV_2_→V_2_EV_2_ titration above the *T*_t_ (T = 60°C). (d) Binding isotherm and fit. (e) Final snapshot of the V_2_EV_2_‐V_2_KV_2_ mixture below the *T*_t_ (T = 20°C) showing spatial separation and solvation shells (Na^+^, Cl^−^ identified). (f) Final snapshot at 60°C showing compacted, interacting IDPPs and ion redistribution. (g) Radius of gyration (*R_g_*) for V_2_EV_2_ and V_2_KV_2_ below the *T*_t_ (T = 20°C) indicating limited structural collapse. (h) *R_g_* above the *T*_t_ (T = 60°C), showing ∼4°A decrease, consistent with thermal condensation.

ITC experiments were performed by titrating V_2_EV_2_ into V_2_KV_2_ and vice versa in water, both below and above the *T*_t_ of the mixture (Figure 3, Figures S12 and S13). Above the *T*_t_, both titrations displayed a sharp decay of the calorimetric signal with molar ratio, indicative of abrupt multivalent interactions within the two‐component condensate. Fitting of the binding isotherms (see Experimental Section) enabled extraction of the apparent stoichiometry *n*, binding constant *K*, and enthalpy (ΔH) for both titration directions. For V_2_EV_2_→V_2_KV_2_, the best‐fit values were ∆H = (8.8 ± 1.2) × 10^5^ J mol*^−^* ^1^ (210.33 ± 28.7 kcal mol*^−^* ^1^), *K* = (4.1 ± 2.1) × 10^9^ M*^−^* ^1^, and *n* = 0.55 ± 0.12. For V_2_KV_2_→V_2_EV_2_, the fitted parameters were ∆H = (5.4 ± 1.7) × 10^5^ J mol^−1^ (129.06 ± 40.6 kcal mol^−1^), *K* = (9.1 ± 1.8) × 10^9^ M*^−^* ^1^, and *n* = 1.36 ± 0.20. In other words, although both titrations exhibit strong apparent affinities and similar enthalpic signatures, the effective stoichiometries diverge markedly. Condensation requires approximately twice as much V_2_KV_2_ as V_2_EV_2_ to reach saturation, in line with the enthalpy maximum shifted toward V_2_KV_2_‐rich mixtures observed by DSC.

The magnitudes of ∆H are substantially larger than typical values reported for IDP‐IDP complexes (0.1–10 kcal·mol^−1^) and coacervates based on oppositely charged polypeptide (0.2–0.7 kcal·mol^−1^) [49, 50, 51]. These large values should not be interpreted as single binding events. Instead, they reflect the cumulative, multivalent reorganization of hundreds of weak, transient interactions together with extensive water and counterion release during LCST‐driven condensation. Consistent with this interpretation, condensation remains primarily entropy‐driven, with the favorable entropy gain outweighing the substantial enthalpic cost of dehydration.

To visualize this collective molecular reorganization and connect thermodynamics with nanoscale structure, atomistic MD simulations of equimolar V_2_EV_2_ + V_2_KV_2_ mixtures were performed at temperatures just below and above the *T*_t_ (Figure 3e–h and Figure S14). Below the *T*_t_, both IDPPs remained solvated and spatially separated, with intact ion clouds of Na^+^ and Cl*^−^* surrounding the chains, especially around the charged side chains. In contrast, above the *T*_t_, the frame showed tight interchain association and partial expulsion of counterions from the condensed chains. This reorganization coincided with a ∼0.4 nm decrease in the radius of gyration (*R_g_*) for both chains (Figure 3g,h and Figure S14g), indicating thermally induced compaction consistent with the ITC results. The concomitant decrease in solvent‐accessible surface area (SASA) further confirmed the loss of the solvation shell and the burial of hydrophilic and hydrophobic patches alike (Figure S14h).

Although ITC and MD are performed under different ionic environments, they reveal complementary aspects of the same LLPS process. ITC measurements, conducted without added salt, quantify the intrinsic bulk behavior driven by the native charge complementarity of V_2_EV_2_ and V_2_KV_2_. In contrast, atomistic MD simulations necessarily include Na^+^ and Cl^−^ to maintain electrostatic neutrality under periodic boundary conditions, providing access to the nanoscale mechanisms that cannot be resolved calorimetrically. The simulations reproduce molecular features consistent with the asymmetry inferred from ITC, including differential hydration, distinct counterion coordination, chain compaction, and enhanced interchain contacts at elevated temperature, despite electrostatic screening by dissolved ions. Spontaneous condensation emerges in the simulations through cooperative desolvation, selective counterion release, chain compaction, and persistent differences in effective charge exposure. These observations support that the thermodynamic driving forces for condensation are not abolished by ionic stabilization. Instead, they suggest a robust condensation regime in which entropy gained from water and counterion release dominates the free energy landscape.

The molecular origin of this asymmetry can be rationalized in terms of sequence‐encoded solvation differences between acidic and basic side chains [43]. Radial distribution functions reveal a pronounced preference of Na^+^ ions for the negatively charged carboxylates of V_2_EV_2_, leading to tighter hydration shells and more effective electrostatic screening (Figure S14e,f). In contrast, Cl^−^ ions associate more weakly with the protonated ammonium groups of V_2_KV_2_, resulting in partial exposure of cationic side chains. This differential ion coordination establishes distinct local solvation environments and generates an unequal electrostatic potential across the two polypeptides. Consequently, V_2_EV_2_ remains more tightly hydrated and locally screened within the condensate, whereas V_2_KV_2_ retains a higher effective positive valence. While the simulations do not model a multichain condensate or a macroscopic phase transition, they support a molecular‐level interpretation in which unequal ion screening contributes to the experimentally observed requirement of excess V_2_KV_2_ to achieve charge compensation in the condensed phase.

Importantly, condensation is accompanied by a sharp increase in the number of interchain hydrogen bonds between V_2_EV_2_ and V_2_KV_2_, paralleling the endothermic transitions observed in ITC (Figure S14). These hydrogen‐bonding interactions likely contribute to stabilizing the condensed state but operate in concert with the dominant entropic gain arising from water and counterion release. In parallel, intramolecular hydrogen bonds become more stable and frequent above *T*_t_, particularly within V_2_EV_2_, reinforcing its tendency to adopt compact, globular conformations upon condensation. This intrachain reorganization further contributes to the observed reduction in *R_g_* and the stabilization of the condensed state.

Snapshots extracted from simulations (Figure 3e,f) illustrate a clear transition from a dispersed, solvated state to a compact, aggregated configuration upon heating above *T*_t_. In particular, clustering of V_2_EV_2_ and V_2_KV_2_ chains is accompanied by visible depletion of surrounding counterions, consistent with a release of solvation constraints—a process expected to contribute positively to the net entropy change upon complexation. The progressive reduction in *R_g_* for both IDPPs, (Figure 3g,h), reinforces the calorimetric interpretation. The ∼0.4 nm decrease observed for both chains was temporally correlated with the formation of stable hydrogen‐bonded contacts and reduced solvent accessibility, suggesting that the macromolecular compaction is not merely entropically favored, but also structurally stabilized [52]. This nanoscale structural reorganization is a hallmark of the phase separation and confirms that the calorimetric signals captured in ITC reflect real molecular rearrangements within the condensate.

### 2.5 pH‐Driven LLPS Reveals Opposite Responses in Charged IDPP Condensates

**FIGURE 4:**
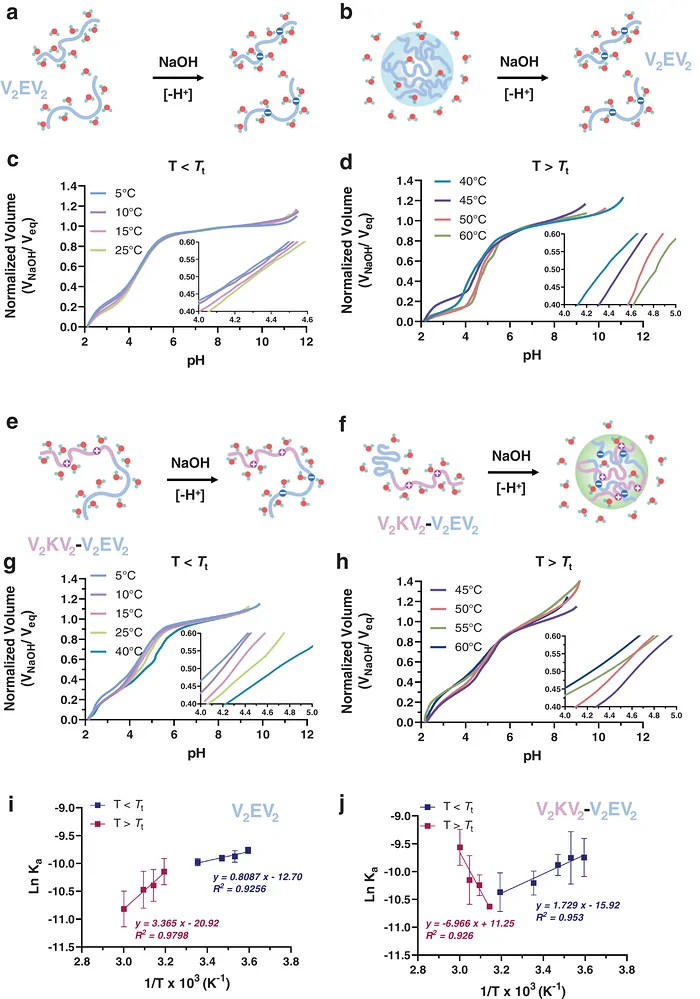
Thermodynamic characterization of the LLPS of V_2_EV_2_ and V_2_KV_2_‐V_2_EV_2_ by acid–base titration above and below the *T*_t_. Schematic representations of the phase behavior and protonation state of (a, b) V_2_EV_2_ or (e, f) V_2_KV_2_‐V_2_EV_2_ titrated below (a, e) or (b, f)) above their *T*_t_. V_2_EV_2_ undergoes condensation only when it is protonated (uncharged, below pK_a_) and heated above *T*_t_, whereas V_2_KV_2_‐V_2_EV_2_ remains soluble at high temperature until deprotonation triggers LLPS. (c, d, g, h) Representative titration curves for V_2_EV_2_ (c, d) and V_2_KV_2_‐V_2_EV_2_ (g, h) monitored across multiple temperatures below (c, g) and above (d, h) the *T*_t_. Insets zoom into the pK_aE_ region (0.5 V_eq_), highlighting the shift in protonation equilibria upon phase separation. (i–j) Van't Hoff plots constructed from titration equilibrium constants at different temperatures for V_2_EV_2_ (i) and V_2_KV_2_‐V_2_EV_2_ (j), showing linear relationships that allow estimation of apparent ΔH° and ΔS° values. V_2_KV_2_‐V_2_EV_2_ exhibits an inverse enthalpic trend compared to V_2_EV_2_ above the LCST, consistent with a condensation mechanism driven by charge neutralization and hydrophobic collapse in the diblock architecture.

To examine whether dense‐phase chemistry influence the acid‐base equilibria in charged IDPPs, we quantified the apparent pK_aE_ values across temperatures below and above *T*_t_. We focused on two pH‐responsive condensate systems that respond oppositely to the same pH stimulus: V_2_EV_2_ and V_2_KV_2_‐V_2_EV_2_ (Figure 4). In V_2_EV_2_, Glu protonation reduces electrostatic repulsion and promotes condensation, whereas deprotonation restores charge and favors solubilization. In V_2_KV_2_‐V_2_EV_2_, by contrast, Glu deprotonation enables Lys–Glu ion pairing and promotes condensation of the diblock.

Acid‐base titrations provide an experimental readout on the apparent pK_aE_ of Glu residues under different phase states (i.e., fully solvated in dilute solution or embedded within a dense phase). Van't Hoff analysis was then used to estimate apparent thermodynamic parameters (ΔH° and ΔS°) of the ionization process (Figure 4i,j). The van't Hoff plots revealed two regimes depending on the phase state, rather than a single linear relationship across the full temperature range. We therefore extracted apparent ΔH° and ΔS° values separately below and above *T*_t_. This behavior is not unexpected, because Glu ionization occurs in two distinct physical environments: soluble, highly hydrated chains below *T*_t_ and a polymer‐rich dense phase above *T*_t_, where hydration, polarity, counterion activity, and polymer–polymer contacts are altered.

Below the *T*_t_, both systems remained soluble and showed systematic increase in pK_aE_ with temperature (Table S3) with ΔH° < 0, ΔS° < 0 (Table S4). This is consistent with strong hydration of the carboxylate anions and counterion binding in bulk solution [42]. The formation of the carboxylate is exothermic because hydration of the anion and association of counterions release heat, yet it is entropically penalized by ordering of water and ions. The increase of pK_aE_ with temperature reflects the reduced stabilization of the deprotonated state, as the dielectric constant of water decreases and dehydration of charged groups becomes more costly, rendering the acid effectively weaker in bulk solution.

Above the *T*_t_, the two systems diverged. V_2_EV_2_ maintained the same qualitative trend (i.e., pK_aE_ increased with temperature and ΔH° < 0, ΔS° < 0), but the deprotonation now drove condensate dissolution (Figure 4b). In this case, Glu ionization breaks intermolecular contacts within the dense state and rehydrates exposed carboxylates, adding energetic and entropic penalties relative to the soluble regime. Importantly, although the signs of ΔH° and ΔS° remained negative, their absolute values were larger above *T*_t_ than below it (Table S4). This reflects the extra energetic cost and entropic penalty associated with breaking intermolecular contacts inside the condensate and rehydrating side chains, together with the restructuring of the solvent shell around newly exposed carboxylates and counterions.

On the other hand, V_2_KV_2_‐V_2_EV_2_ exhibited the opposite response above the *T*_t_. Its apparent pK_aE_ decreased with increasing temperature (Table S3), and both ΔH° and ΔS° became positive (Table S4). Here, deprotonation is endothermic (enthalpically costly) but entropically favorable, indicating that the release of water and counterions and the formation of stabilizing salt bridges dominates the free‐energy balance (Figure 4f,h). At acidic pH (pH < pK_aE_), the Glu residues remain protonated, and therefore the V_2_EV_2_ blocks tend to collapse. However, V_2_KV_2_‐V_2_EV_2_ remained soluble because of the highly solvated cationic V_2_KV_2_ block, which prevents condensation. As the pH increased (pH > pK_aE_), deprotonated Glu‐COO^−^ engaged in electrostatic pairing with Lys‐NH_3_^+^ residues, promoting salt‐bridge formation, water release, and ultimately hydrophobic collapse of the diblock copolymer.

The contrast between both systems is reflected in their apparent enthalpies above *T*_t_. V_2_EV_2_ showed a negative ΔH° = −6.7 kcal·mol^−1^, consistent with deprotonation coupled to hydration and condensate dissolution. V_2_KV_2_‐V_2_EV_2_ showed a positive ΔH° of 14.9 kcal·mol^−1^, consistent with deprotonation coupled to ion‐pair formation and condensate stabilization. Although chain length contributes to the total enthalpy (V_2_KV_2_‐V_2_EV_2_ contains twice as many pentapeptide repeats) the magnitude of ΔH° arises primarily from the cooperative reorganization of its diblock architecture. The simultaneous formation of salt‐bridge networks and enhanced hydrophobic contacts amplifies the energetic cost of ionization while stabilizing the condensed state, consistent with the stronger reversing signal observed in TM‐DSC (Figure 2f). Once two phases coexist in charged condensate systems, ions may distribute unequally between the dense condensate and dilute phases [21, 22]. Such ion partitioning might create an electrostatic potential difference between the two phases, which defines a distinct internal pH and ion activity inside the condensate. Because the apparent pK_aE_ of ionizable residues is referenced to the external pH, this internal potential effectively shifts the measured pK_aE_ values within the dense phase and can even invert the temperature dependence observed for V_2_EV_2_ and V_2_KV_2_‐V_2_EV_2_. In parallel, changes in the microenvironment of the condensate, i.e., changes in effective dielectric constant, hydration, and viscoelastic network [27, 53], also influence which ionization state is stabilized [22, 53].

These combined effects delineate two pH‐responsive routes to condensation. V_2_EV_2_ exemplifies a protonation/neutralization pathway, where acidification reduces electrostatic repulsion, increases backbone proximity, and promotes water release, stabilizing the condensed state. This mechanism simulates the behavior of native acidic IDPs, such as the RPT segment of Pmel17 [54], which is soluble at cytosolic pH yet solidifies into functional amyloid upon melanosomal acidification [55]. In contrast, V_2_KV_2_‐V_2_EV_2_ exemplifies a deprotonation/ion‐pairing pathway. Above the *T*_t_, a cation‐rich, lower‐dielectric interior favors Glu^−^‐Lys^+^ pairing, thus lowering the dense‐phase pK_aE_, and reinforces LLPS despite the endothermic cost of ionization.

### 2.6 Intramolecular Salt‐Bridges Encode Condensate Microenvironment

To connect salt‐bridge topology to the condensate microenvironment and miscibility, we combined NMR reporters of the local electrostatic environment of Lys residues with confocal microscopy to visualize mixing behavior in the presence of a non‐charged partner (V). Here, NMR is used to infer the apparent spectral populations associated with distinct Lys electrostatic environments, which are compatible with different extents and topologies of ionic pairing and may modulate the apparent residual free charge within the dense phase.

**FIGURE 5:**
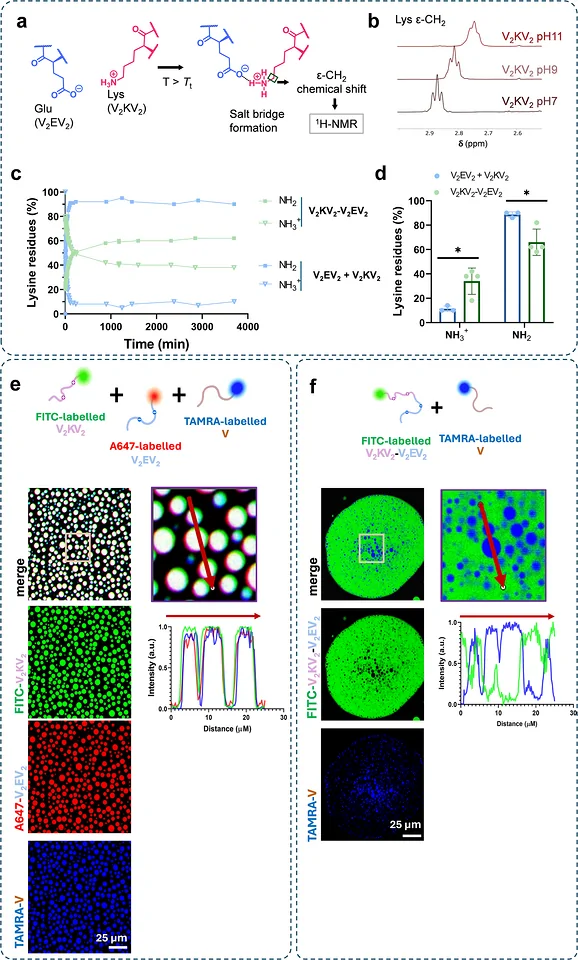
Salt‐bridge topology modulates condensate microenvironment and partitioning behavior in IDPP condensates. (a) Schematic representation of electrostatic pairing between glutamic acid and lysine side chains, highlighting salt‐bridge formation through carboxylate‐ammonium interaction. (b) Representative ^1^H‐NMR spectra of V_2_KV_2_ at pH 7, 9, and 11. The chemical shift of the ε‐methylene protons (‐CH_2_‐) adjacent to the terminal amine group (Lys) shift upfield with increasing pH, consistent with reduced local positive charge and Lys deprotonation. This signal was used as a reporter for changes in the local electrostatic environment sensed by Lys residues. (c) Time‐resolved evolution of the apparent Lys spectral populations assigned to less screened (NH_3_^+^) and more electrostatically screened environments (NH_2_) in V_2_KV_2_ + V_2_EV_2_ and V_2_KV_2_–V_2_EV_2_ condensates during LLPS. (d) Final apparent screened (NH_2_)/ less screened (NH_3_^+^) ratios after 3000 min. V_2_EV_2_ + V_2_KV_2_ shows a significantly higher apparent screened Lys population, consistent with more extensive intermolecular charge compensation. (e,f) Confocal microscopy images of condensates containing a third, neutral hydrophobic IDPP (V), mixed either with V_2_KV_2_ + V_2_EV_2_ (e) or with V_2_KV ‐V_2_EV_2_ (f). In V_2_KV_2_ + V_2_EV_2_, all components colocalize uniformly throughout the condensates. In contrast, in V_2_KV_2_‐V_2_EV_2_ condensates, changes in polarity lead to the formation of discrete immiscible V‐based microdomains. Fluorescence intensity profiles along representative condensates confirm distinct partitioning behaviors. TAMRA is displayed in blue to improve contrast.

Direct detection of salt‐bridge formation by 2D‐NMR (e.g., ^1^H‐^15^N HMBC and ^1^H‐^1^H NOESY) was attempted but proved not feasible due to severe signal broadening in the condensed phase (Figures S15–S22). This broadening reflects the limitations of the technique in highly crowded protein phases, where restricted molecular motion and rapid transverse relaxation (T_2_) suppress long‐range correlations and exacerbate peak overlap. Although occasional weak cross‐peaks were observed, they could not be unequivocally assigned to specific interactions between Glu and Lys side chains, impeding precise quantification of these contacts (Figure S21). Instead, we monitored the ε‐methylene hydrogens of Lys, adjacent to the terminal primary amine, which serve as sensitive reporters of the local electrostatic environment surrounding the Lys side chain (Figure 5a). At pH 7, these hydrogens produced a resonance at 2.88 ppm and, upon increasing pH, the signal shifted progressively upfield consistent with deprotonation of the Lys ammonium group and a reduction of the local positive charge (Figure 5b).

A similar upfield displacement of the Lys ε‐methylene resonance was observed for V_2_KV_2_ + V_2_EV_2_ mixtures driven above the *T*_t_ in D_2_O, even under controlled bulk‐pH conditions (Figure S23). Because the external pH remains constant, this shift cannot result from simple basification of the medium. We interpret this effect as consistent with a change in the local electrostatic environment of Lys residues, likely due to the upon ionic pairing with Glu–COO^−^ groups and electrostatic charge compensation within the dense phase. This effect reproduces the spectral behavior observed upon basification in bulk solution, but here is assigned to local electrostatic screening within the condensate microenvironment rather than from a change in bulk pH.

This approach enabled us to estimate the apparent fraction of Lys residues experiencing a more electrostatically screeened, ion‐paired‐like environment (Figure 5c,d). In equimolar V_2_EV_2_+ V_2_KV_2_ mixtures, this apparent spectral population reached approximately 90%, consistent with extensive intermolecular charge compensation between the two oppositely charged IDPPs. In the covalently linked diblock V_2_KV_2_‐V_2_EV_2_, this population dropped to ∼70%. We attribute this reduction to the formation of intramolecular salt bridges imposed by the diblock architecture, which might constrain conformational freedom and limit intermolecular pairing. Thus, these percentages should be regarded as apparent populations of Lys electrostatic environments, not as direct percentages of Lys residues forming salt bridges.

These measurements indicate that molecular architecture, either as a mixture of oppositely charged chains or as a covalently linked diblock, determines the electrostatic environment sensed by Lys residues and therefore, the polarity of the condensed phase. Because the condensate microenvironment governs condensate miscibility and client partitioning [27], we hypothesized that differences in apparent charge compensation, i.e., a greater fraction of unpaired ionic groups, would increase micropolarity and thus alter miscibility with other IDPs. To test this, we designed multicomponent condensates by combining the charged systems with a hydrophobic LCST‐IDPP, V. Throughout this study, topology refers not to classical secondary/tertiary structure, but to the network connectivity of ionic contacts imposed by sequence architecture.

In the presence of IDPP V, both systems (equimolar V_2_EV_2_ + V_2_KV_2_ and covalently linked V_2_KV_2_‐V_2_EV_2_) underwent LLPS, concentrating virtually all polypeptides into dense phases (Figure 5e,f). However, their internal organization differed markedly. In V_2_EV_2_ + V_2_KV_2_ + V mixtures, where charge compensation is dominated by intermolecular pairing and the apparent screened Lys population is higher (∼90%), all three IDPPs coacervated into homogeneous spherical condensates, with a uniform distribution throughout the interior (Figure 5e). This is consistent with a lower‐polarity interior compatible with the hydrophobic partner. In contrast, V_2_KV_2_‐V_2_EV_2_ + V mixtures exhibited spatial segregation of V into distinct internal compartments within the V_2_KV_2_‐V_2_EV_2_ condensates (Figure 5f and Figure S24). In this condensate system, the intra‐chain pairing is promoted and the overall fraction of ionic pairing is decreased (∼70%), consistent with a greater porportion of residual free charge within the dense phase. Such internal demixing is in line with the general behavior of multiphase polymer‐rich droplets, where differences in dense‐phase hydration can stabilize sharp internal interfaces and selective partitioning of client components [33].

In this way, these findings establish a direct connection between protein polymer architecture, salt‐bridge topology, and condensate polarity. Extensive intermolecular charge compensation (V_2_EV_2_ + V_2_KV_2_) favors compositionally homogeneous multicomponent condensates, whereas the covalent diblock (V_2_KV_2_‐V_2_EV_2_) promotes a distinct distribution of ionic contacts that preserves a more polar microenvironment and favors internal demixing and multiphase condensation in the presence of a non‐charged IDPP partner. By biasing salt‐bridge formation within a chain (intramolecular) or between chains (intermolecular), the sequence modulates the degree of residual charge in the dense‐phase, thereby influencing condensate miscibility and phase organization.

This architectural control of condensate microenvironment opens a route for bottom‐up biofabrication. By tuning charge patterning, block arrangement, and stoichiometry, the same protein building blocks can generate condensates with programmable compartmentalization. We expect that this system can be used to condense enzymes, cofactors, nucleic acids, and small‐molecule substrates to create, for example, selectively partitioning microreactors that support compartmentalized catalysis, stimuli‐responsive gating, and dynamic and tunable scaffolds for tissue engineering [56, 57]. Such control over micropolarity using the same polypeptide components also enables hierarchical condensate assemblies, reminiscent of the nucleolus in cells and consistent with in vitro polymer systems [31, 33, 58], thereby creating microenvironments with defined micropolarities and thus, biochemical properties. Moreover, the intrinsically charged nature of these condensates means that co‐assembly with ions and small molecules offers a second means of controlling dense‐phase properties [53, 59]. Hence, future work will focus on, systematically varying ionic identity, strength, and cargos of clinical relevance to reveal how these interactions tune internal organization, micropolarity gradients and functionality.

## 3 Conclusion

In summary, we establish a sequence‐encoded framework for programming the phase behavior, thermodynamic landscape, and microenvironmental properties of synthetic biomolecular condensates using a minimal, genetically encoded LCST‐IDPP platform. By comparing sequence‐matched oppositely charged IDPPs and varying chain architecture (binary mixture vs. covalently linked diblock), we reveal how the interplay between hydrophobic collapse and electrostatic interactions governs the onset, cooperativity, and internal organization of bicomponent IDPP condensates. Combining calorimetry, spectroscopy, and molecular simulations, we uncover two distinct energetic regimes (i.e., slow cooperative dehydration and rapid chain reorganization) that emerge from the interplay between intra‐ and intermolecular salt‐bridge formation, solvent reorganization, and chain desolvation. The results reveal that the topology of ionic interactions, whether encoded as interchain or intrachain pairings, determines the apparent residual charge and micropolarity of the dense phase and thereby controls the miscibility of condensates with additional coacervation partners. These findings provide a quantitative and mechanistic framework to rationally design synthetic condensates with programmable phase behavior and micropolarity, offering a minimal model to interrogate how sequence and connectivity encode multiphase organization. Beyond their fundamental relevance, these insights open new avenues for engineering responsive microenvironments, selective molecular partitioning, and dynamic scaffolds for catalysis, biosensing, and tissue engineering.

## 4 Experimental Section

### 4.1 Production and Characterization of the IDPP Library

The IDPPs were produced recombinantly in *Escherichia coli* following previously described methods [60]. Briefly, the encoding genes were purchased from NZYTech (Portugal) in the pUC57 cloning plasmid, subcloned into an expression vector derived from pET25(+), and transformed into *E. coli* BLR(DE3). Recombinant expression was carried out by bacterial fermentation in a 15 L bioreactor (Applikon) using modified Terrific Broth (TB) medium under continuous stirring at 500 rpm and 37°C. After 18 h of fermentation, the cells were harvested by centrifugation at 4°C and washed with isotonic buffer to remove residual fermentation components. The cell pellets were then lysed by disruption (GEA Panda 2000).

Purification of the IDPPs was achieved through their LCST phase behavior using inverse transition cycling [61]. The purified polypeptides were subsequently dissolved and dialyzed extensively against 25 L of water (type II, three changes and type I, one change) at 4°C, sterilized by filtration (0.22 µm, Nalgene), and lyophilized (Labconco). The pH of the dialyzed polymer solutions was measured immediately before lyophilization (Table S1). These values were taken as the apparent isoionic pH of each polymer preparation. Typical yields ranged from 530 to 750 mg of purified polymer per liter of culture medium.

The monodispersity and purity of the IDPPs were evaluated by sodiumdodecyl sulfate‐polyacrylamide gel electrophoresis (SDS‐PAGE), matrix‐assisted laser desorption/ionization time‐of‐flight (MALDI‐ToF) mass spectrometry, high‐performance liquid chromatography (HPLC), and NMR. The MALDI‐ToF, HPLC, and NMR experiments were carried out at the Laboratory of Instrumental Techniques (LTI) Research Facilities, University of Valladolid.

### 4.2 Chemical Functionalization for Confocal Microscopy

Fluorescent derivatives of the IDPP library were prepared by covalent conjugation of fluorescent probes bearing amine‐reactive groups, i.e., N‐hydroxysuccinimide (NHS), to primary amines via nucleophilic substitution. Briefly, solutions of the dyes (i.e., Alexa Fluor 647 NHS ester, fluorescein isothiocyanate (FITC), and 5(6)‐carboxytetramethylrhodamine N‐succinimidyl ester (TAMRA‐SE) (all from Thermo Fisher Scientific)) were dissolved in anhydrous DMSO and mixed with IDPP solutions in anhydrous DMF at a 1:1 molar ratio (dye:IDPP). The reaction mixtures were stirred for 24 h at room temperature under a nitrogen atmosphere. After completion, the products were dialyzed repeatedly against 5L of water (three changes in type II and two changes in type I) at 5°C, sterilized by filtration through 0.22 µm membranes, and lyophilized.

For all confocal microscopy experiments, the fluorescently labelled IDPPs were mixed with their unlabeled counterparts to ensure that 10 mol% of the total polymer in solution was labelled, thereby minimizing perturbation of the native phase‐separation behavior.

### 4.3 Condensate Visualization Setup

For visualization, 70 µL of IDPP solution was placed on a glass slide (Fisherbrand, 12373118) equipped with an incubation chamber composed of an adhesive spacer frame (Frame‐Seal BIO‐RAD, SLF0601) and a cover glass (Epredia, 15747592) positioned on top of the spacer.

Both the slide and the cover glass were subjected to a Pluronic F‐127 (Sigma–Aldrich) treatment to prevent surface adhesion, creating a non‐stick layer on the glass surfaces to inhibit the attachment of the IDPPs [62]. This treatment consisted of cleaning with 70% (v/v) ethanol, followed by rinsing with (type I) water and air‐drying under a laminar flow hood. The components were then immersed in a freshly prepared 5% (v/v) Pluronic F‐127 solution for 1 h at room temperature. Unbound Pluronic was subsequently removed by washing ten times (1 min per wash) with type I water. Finally, the treated components were air‐dried in a sterile environment before use.

### 4.4 Confocal Microscopy

Fluorescence images were acquired using a TCS SP8 confocal microscope (Leica Microsystems, Germany) equipped with a 405‐nm laser and a pulsed white light laser (WLL). Imaging was performed with a 40x oil‐immersion objective (HC PL Apo 40x/1.30oil CS2) integrated with an environmental heating chamber for temperature control. Excitation wavelengths were set to 491 nm for FITC, 552 nm for TAMRA, and 653 nm for Alexa Fluor 647.

Prior to imaging, IDPP solutions were prepared at a final concentration of 250 µm in type I water and incubated within the sealed cover‐glass assembly at 60°C for 20 min to induce LLPS. For multiphase evaluation, the components were mixed while maintaining a constant total IDPP concentration of 250 µm, corresponding to 125 µm of each block. Image acquisition and processing were performed using Leica Application Suite X (LAS X) software.

Colocalization analysis was performed using ImageJ on the two fluorescence channels acquired for the binary system. Pearson's correlation coefficient and Manders split coefficients were calculated using the Coloc2 plugin.

### 4.5 Evaluation of the Thermal Behavior by Turbidimetry

Turbidimetry experiments were conducted using a Varian Cary 100 UV–vis spectrophotometer (Varian Inc., NC, USA) equipped with a temperature‐controlled cuvette holder. The optical density at 350 nm (OD^350^) of the IDPP solutions was recorded under different conditions.

Two sets of experiments were carried out after dissolving the IDPPs in type I water, used as a non‐buffered low‐ionic‐strength aqueous medium without externally added salts, or in PBS buffer. In the first set, a concentration series of IDPPs, including a physical mixture of V_2_EV_2_ and V_2_KV_2_ (V_2_EV_2_ + V_2_KV_2_), was prepared at 10, 15, 25, 50, 75, 100, 150, 200, 300, 500, and 1000 µm. In the second set, the influence of charge stoichiometry on coacervation was investigated by mixing V_2_EV_2_ and V_2_KV_2_ at different volumetric ratios (100:0, 80:20, 60:40, 50:50, 40:60, 20:80, 0:100) at a fixed final concentration of each IDPP of 25 µm. All data were collected at a scan rate of 1°C min^−1^, and each condition was analyzed in triplicate. The transition (cloud point) temperature (*T*_t_) was determined as the temperature corresponding to Programming molecular self‐assembly of intrinsically disordered proteins containing sequences of low complexity the maximum of the first derivative of the OD^350^ vs. temperature curve.

### 4.6 Droplet Formation

Water‐in‐oil emulsion droplets were generated using a PDMS microfluidic droplet generator with a flow‐focusing geometry (Droplet Generator‐PDMS‐DG‐CF‐60/1.2 M SK: DG‐CF‐60‐12 Darwin Microfluidics). The system consisted of two immiscible liquid phases. The dispersed aqueous phase contained IDPPs at a total concentration of 250 µm in type I water. The continuous oil phase consisted of TEGOSOFT DEC/ABIL EM 90/mineral oil at a 75/5/20 volumetric ratio, respectively, as previously described [31]. Both phases were injected into the device using high‐precision syringe pumps (pump 11 Elite, Harvard Apparatus) at room temperature. The continuous phase was injected at 250 µL h^−1^, while the aqueous phase was maintained at 50 µL h^−1^.

### 4.7 Enthalpy Analysis by DSC and TM‐DSC

DSC and TM‐DSC experiments were performed using a Mettler Toledo 822e calorimeter equipped with a liquid nitrogen cooling system. IDPP solutions (50 mg mL^−1^) were prepared in type I water at different pH values and in PBS buffer. In addition, mixtures of V_2_EV_2_ and V_2_KV_2_ at the same volumetric ratios used in the turbidimetry experiments were analyzed under identical conditions. For experiments in which the pH was adjusted to 7, the lyophilized polymers or polymer mixtures were first dissolved in type I water and then corrected with small amounts of NaOH or HCl. Under these concentration conditions, the estimated ionic contribution introduced by pH adjustment remained below 2 mm for the equimolar V_2_EV_2_ + V_2_KV_2_ mixture and for the diblock V_2_KV_2_‐V_2_EV_2_. For the individual charged IDPPs, this contribution was higher, reaching approximately 5 mm NaOH for V_2_KV_2_ and 10 mm HCl for V_2_EV_2_, with intermediate values for the off‐stoichiometric mixtures.

For all experiments, 20 µL of each prepared solution was introduced into a standard 40 µL aluminium pan, which was hermetically sealed. An equivalent volume of solvent was added to a reference pan.

In conventional DSC experiments, samples were equilibrated for 2 min at 20°C and subsequently heated at a constant rate of 2°C min^−1^. TM‐DSC measurements were conducted by superimposing a sinusoidal temperature modulation onto a linear heating ramp. The modulation parameters were: underlying heating rate (ν) = 1°C min^−1^, amplitude (A) = 0.1°C, and period (P) = 0.6 min. The selected TM‐DSC conditions correspond to those previously optimized for the analysis of this class of IDPPs, ensuring reliable deconvolution of the reversing and non‐reversing enthalpic components [38, 63].

### 4.8 Evaluation of the Secondary Structures by Circular Dichroism (CD) Spectroscopy

The secondary structures of the IDPP library and mixtures from the V_2_EV_2_ + V_2_KV_2_ system were analyzed by CD spectroscopy using a Jasco J‐810 spectropolarimeter (Jasco, U.S.A.) equipped with 0.1 cm quartz cells and with a temperature controller (STTI, University of Alicante, Spain). Samples were prepared at a concentration of 0.35 mg mL^−1^ in type I water, and the pH was adjusted to 7. Experiments were conducted below and above the *T*_t_, specifically at 20°C and 60°C, with a stabilization time of 20 min prior to data acquisition. CD spectra were recorded in the wavelength range of 190–250 nm. The data were smoothed using a 15‐point Savitzky–Golay filter.

The secondary‐structure content of each sample was estimated from the experimental spectra using the BeStSel algorithm [64, 65], which enables quantitative deconvolution of β‐sheet, α‐helix, and disordered contributions in proteins.

### 4.9 Analysis of the Thermodynamic Parameters of Interactions by ITC

The binding affinity and stoichiometry governing coacervation of V_2_EV_2_ and V_2_KV_2_ were determined below (22°C) and above (60°C) the *T*_t_ using a MicroCal VP‐ITC microcalorimeter (Malvern) at the Soft Materials Service – U6 NANBIOSIS, Institute of Materials Science of Barcelona (CSIC, Spain).

To probe the directionality of binding, two titration schemes were conducted at each temperature: (1) V_2_EV_2_ in the calorimetric cell and V_2_KV_2_ in the syringe, and (2) V_2_KV_2_ in the cell and V_2_EV_2_ in the syringe. Both IDPPs were dissolved in type I water to minimize baseline heat from buffer mismatch. The concentration in the cell was 57.9 µm, and in the syringe 579.6 µm. Each titration consisted of 35 injections of 9 µL. Control titrations (V_2_KV_2_ or V_2_EV_2_ into type I water) were performed to account for dilution heats, which were subtracted from the raw data to obtain the net heat of binding.

The theoretical model used for fitting is based on the standard equilibrium binding formalism. The fraction of occupied binding sites, θ, is defined in terms of the equilibrium constant K and the free ligand concentration ([X]) by the equation:

$$K=\frac{\theta }{\left(1-\theta \right)\left[X\right]}$$

This relationship is coupled with the conservation of mass, which for a macromolecule of total concentration Mt and a ligand of total concentration Xt, takes the form:

$$Xt=\left[X\right]+n\theta Mt$$

Combining the two expressions yields a quadratic equation in θ:

$$\theta^{2}-\theta \left(1+\frac{Xt}{n\cdot Mt}+\frac{1}{n\cdot K\cdot Mt}\right)+\frac{Xt}{nMt}=0$$

Once θ is solved at each injection point, the total heat content Q of the cell at fractional saturation is calculated as:

Q=n·Mt·ΔH·VO·θ

where ΔH is the molar binding enthalpy and V0 is the cell volume. However, since titration involves the addition of finite aliquots of ligand solution, a correction must be applied for the displaced volume after each injection. This correction accounts for the fact that a portion of the solution previously in the cell is ejected with each addition, yet still contributes to the observed heat signal due to fast mixing and reaction kinetics. The corrected heat evolved after the ith injection, ΔQ(i), is then computed as:

$$\Delta Q\left(i\right)=Q\left(i\right)+\frac{\delta Vi}{V0}\left[\frac{Q\left(i\right)+Q\left(i-1\right)}{2}\right]-Q\left(i-1\right)$$

This expression was applied iteratively during nonlinear regression fitting, using a Marquardt‐Levenberg algorithm to minimize the residuals between experimental and calculated heats. The parameters of interest—stoichiometry n, binding constant K, and enthalpy ΔH—were extracted for both titration directions.

### 4.10 Computational Methods

Atomistic MD simulations were conducted to resolve the nanoscale structural rearrangements underlying thermoresponsive coacervation in mixtures of V_2_EV_2_ and V_2_KV_2_. Initial chain conformations were generated using AlphaFold2, which predicted highly flexible structures with limited tertiary organization [66], consistent with the expected behavior of IDPPs with LCST behavior (i.e., elastin‐like recombinamers, ELRs). For V_2_EV_2_, the predicted structural metrics included ptm values 0.16–0.26 and fraction of disorder ∼0.25. V_2_KV_2_ exhibited ptm ≈ 0.24 and ∼0.21 disorder, with low chain‐pair confidence (chain_pair_iptm ≈ 0.24). These modest confidence values reflect the inherent conformational heterogeneity of disordered polypeptides and do not indicate structural inaccuracies. Instead, they validate that both ELRs exist as dynamic ensembles lacking rigid folded domains, a prerequisite for reversible coacervation.

The predicted structures were placed in a cubic periodic box of explicit water (TIP3P model), and Na^+^ and Cl^−^ ions were added to ensure overall charge neutrality and prevent unphysical intrachain electrostatic repulsion under periodic boundary conditions. An equimolar 1:1 mixture of V_2_EV_2_ and V_2_KV_2_ was simulated to probe the onset of intermolecular associations driving condensation.

All simulations employed the CHARMM36m all‐atom force field, optimized for IDPs, along with CHARMM parameters for monovalent ions. Long‐range electrostatic interactions were evaluated using particle mesh Ewald (PME) with a 10–12 Å real‐space cutoff. Bond constraints involving hydrogens were enforced with LINCS, allowing a 2 fs time step. Temperature and pressure were controlled using the Nosé–Hoover thermostat and Parrinello–Rahman barostat at 20°C (below the transition temperature) and 60°C (above *T*_t_), and 1 bar, respectively.

All systems underwent energy minimization, followed by NVT equilibration (200–500 ps) and NPT equilibration (1–2 ns), prior to production runs of 40 ns per temperature condition. Analyses were performed using GROMACS and VMD, quantifying the radius of gyration (*R*_g_), solvent‐accessible surface area (SASA), inter‐ and intrachain hydrogen bonding, and radial distribution functions (RDFs) between charged residues and counterions. Temporal correlations of these features enabled evaluation of thermally induced compaction, hydration‐layer depletion, and ion redistribution within emerging protein‐rich clusters.

These MD simulations were not intended to reproduce absolute calorimetric values—ITC experiments were performed without added buffer salts—but instead to provide a mechanistic interpretation of the entropy‐driven coacervation detected experimentally. The emergence of compositional asymmetry and spontaneous interchain association even under electrostatic screening confirms that counterion and water release, rather than mere Coulombic pairing, dominate the driving forces behind condensate formation in this two‐component IDPP system.

### 4.11 Determination of Apparent pK_a_ Values by Acid‐Base Titration

Acid–base titrations were performed using a TitroLine 7000 automatic titrator (SI Analytics, Germany) equipped with a 20 mL burette and an A162 2M‐DIN combined glass electrode. The sample temperature was controlled by connecting a refrigerated circulating bath to a double‐jacketed reaction flask (Merck), ensuring continuous water flow through the system during measurements.

Before each experiment, the pH electrode was calibrated at the measurement temperature using standard buffer solutions. A three‐point calibration was automatically executed by the instrument to guarantee precision and stability across the full titration range.

For each titration, samples were dissolved in type I water to a final IDPP concentration of 2.5 mm in a final volume of 5 mL. The sample mass was adjusted to maintain an equivalent total number of carboxylic groups across all systems, allowing direct comparison of ionizable site behavior. The initial pH was adjusted to approximately 2.0 by incremental addition of 0.1 M HCl.

Titrations were carried out under continuous magnetic stirring at 500 rpm using the instrument's control software. A 55 mm NaOH solution was delivered from the burette at a constant flow rate of 50 µL·min^−1^ until the pH reached approximately 11.0. The pH and titrant volume were recorded in real time throughout the titration.

Apparent pK_a_ values were extracted from the titration profiles as the midpoint of the buffer region corresponding to the maximum of the first derivative (dpH/dV). When the inflection point was not sharply defined, pK_a_ values were determined by linear interpolation of the pre‐ and post‐inflection baselines to minimize experimental uncertainty.

### 4.12 Characterization by NMR

NMR experiments at various temperatures were performed on 500 MHZ Agilent DD2 instruments equipped with a cold probe in the Laboratory of Instrumental Techniques (LTI) Research Facilities, University of Valladolid. Chemical shifts for ^1^H, ^13^C, ^15^N nuclei were measured in ppm, with the HDO residual solvent signal used as an internal reference or nitromethane (CH_3_NO_2_).

Two different temperatures (25°C and 60°C) were employed during the 2D NMR studies. Classical 2D NMR methods were employed in order to achieve ^1^H, ^13^C and ^15^N peak assignments employing 500 MHZ Agilent DD2 instruments equipped with a HFX indirect probe or oneNMR probe in the Laboratory of Instrumental Techniques (LTI) Research Facilities, University of Valladolid. For heteronuclei such as carbon‐13 and nitrogen‐15, frequency‐swept (adiabatic) pulses in place of hard 180° pulses were used to improve spectral quality.

Homonuclear ^1^H–^1^H 2D experiments, including total correlation spectroscopy (zTOCSY) and nuclear Overhauser effect spectroscopy (NOESY), were used with a zero quantum filter were used for artifact suppression [67]. zTOCSY experiments were acquired in the phase‐sensitive mode using the PRESAT pulse sequence in order to suppress the residual water signal resonance. A total of four transients for each of the 200 t1 increments were recorded, using a spectral width of 2323.42 Hz for both dimensions (a total resolution of 12 Hz in the indirect dimension), with a mixing time for the DIPSI2 spin lock of 100 ms and an acquisition time of 150 ms. The data were apodized using a Gaussian window in both dimensions. NOESY experiments were performed using the PRESAT pulse sequence to remove the residual water signal resonance, with the following acquisition parameters: 16 transients for each of the 400 t1 increments, a spectral width of 2323.42 Hz in both dimension (a total of 6 Hz resolution in indirect dimension), and an acquisition time of 150 ms. A short mixing time of 200 ms was used to minimize undesirable spin‐diffusion effects [68]. Data were fitted with Gaussian window in both dimensions. Carbon signals were measured indirectly using an ^1^H‐^13^C HSQC and HMQC experiments. They were acquired with inverse detection and carbon decoupling during acquisition in the phase‐sensitive mode, with PRESAT solvent suppression, employing the following acquisition parameters: 64 transients for each of the 128 t1 increments, 2323.42 Hz spectral width in F2 dimension and 8547.12 Hz in the F1 dimension (a total of 67 Hz resolution in indirect dimension) and 146 Hz as a nominal value for the one‐bond coupling constant J_CH_. Data were fitted with Gaussian window in both dimensions. Nitrogen signals were obtained indirectly using ^1^H‐^15^N HMBC sequence in the phase‐sensitive mode with PRESAT solvent suppression, employing the following acquisition parameters: 512 or 800 transients for each of the 30 t1 increments, 2385.5 Hz spectral width in F2 dimension and 12158.1 Hz in the F1 dimension (a total of 405 Hz resolution in ^15^N indirect dimension) and 3 or 5 Hz as a nominal value for the long‐range coupling constant nJNH. Data were fitted with sinebell window in F2 dimension and Gaussian window in F1 dimension.

All spectra were post‐processed and analysed using Mestrelab Research software (MNova 15.0) and VnmrJ4.2 software (Agilent).

### 4.13 Statistical Analysis

All experiments were performed with at least three independent replicates unless otherwise stated. Data are presented as mean ± standard deviation (SD). Statistical analyses were performed using GraphPad Prism 8. For calorimetric analyses, sample sizes were as follows: TM‐DSC (*n* = 3); DSC in type I water: V (*n* = 7), V–V (*n* = 5), V_2_KV_2_‐V_2_EV_2_ (*n* = 6), V_2_EV_2_ + V_2_KV_2_ mixtures (*n* = 7), V_2_EV_2_ (*n* = 3), and V_2_KV_2_ (*n* = 3); DSC in PBS: V (*n* = 7), V–V (*n* = 7), V_2_KV_2_– V_2_EV_2_ (*n* = 6), V_2_EV_2_ + V_2_KV_2_ mixtures (*n* = 4–6, depending on the molar ratio), V_2_EV_2_ (*n* = 3), and V_2_KV_2_ (*n* = 3). One‐way ANOVA followed by Tukey's multiple‐comparison test was applied to CD spectroscopy data (BeStSel) to evaluate differences in secondary‐structure content among conditions. For NMR experiments quantifying the apparent fraction of Lys residues electrostatically screened, the percentage was analyzed by two‐way ANOVA followed by Sidak's multiple‐comparison test.

## Funding

This work was funded by the Spanish Government (grants PID2021‐122444OB‐100 and PID2022‐137484OB‐I00 funded by MCIN/AEI/ 10.13039/501100011033 and by ERDF, EU), the Junta de Castilla y León (grant VA188P23 and CLU‐2023‐1‐05 cofunded by ERDF), Centro en Red de Medicina Regenerativa y Terapia Celular de Castilla y León, and Xunta de Galicia (grant IN606B‐2023/006).

## Conflicts of Interest

The authors declare no conflicts of interest.

## Supporting information




**Supporting File**: smll74740‐sup‐0001‐SuppMat.docx.

## Data Availability

The data that support the findings of this study are available from the corresponding author upon reasonable request.
