# Supplementary material for: Topology‐Encoded Charge Polarity Governs Multiphase Organization in Intrinsically Disordered Protein Polymer Condensates
Source: Small. 2026 Jul 28;22(52):e74740. doi: 10.1002/smll.74740 (PMC13580316; doi:10.1002/smll.74740)
Supplement: Supplementary file 1 [file SMLL-22-e74740-s001.docx]

Supporting Information

Topology-Encoded Charge Polarity Governs Multiphase Organization in Intrinsically Disordered Protein Polymer Condensates

Julio Fernández-Fernández^1^, Vicente Domínguez-Arca^2,3^, Raúl Escribano^1^, Sheila Lorenzo^1^, Sergio Ferrero^4^, Sergio Acosta^1*^, Jose Carlos Rodríguez-Cabello^1*^

^1^Bioforge Laboratory (Group for Advanced Materials and Nanobiotechnology), Laboratory for Disruptive Interdisciplinary Science (LaDIS), CIBER-BBN, Edificio LUCIA, Universidad de Valladolid, 47011 Valladolid, Spain

^2^Biosystems and Bioprocess Engineering (Bio2Eng) Group, Institute of Marine Research of Spanish Research Council, IIM-CSIC, 36208 Vigo, Spain

^3^Colloids Physical and Biophysical Chemistry, Bielefeld University, Universitätsstr. 25, Bielefeld 33615, Germany

^4^GIR MIOMeT, IU CINQUIMA/Química Inorgánica, Facultad de Ciencias, Universidad de Valladolid, 47011, Valladolid, Spain

*Email: [sergio.acosta@uva.es](mailto:sergio.acosta@uva.es), [carlos.roca@uva.es](mailto:carlos.roca@uva.es)

**Table S1.** Amino-acid sequences, theoretical and experimental molecular weights, theoretical isoelectric points, and apparent isoionic pH of the IDPPs.

| **IDPP** | **Sequence (aa)** | **Theo. Mw^a^**  **(Da)** | **Exp. Mw^b^**  **(Da)** | **Theo.pI^a^** | **Apparent Isoionic pH** | |
| --- | --- | --- | --- | --- | --- | --- |
| V_2_KV_2_ | MESLLP [(VPGVG)_2_VPGKG(VPGVG)_2_]_10_V | 21552.67 | 21545.67 ± 0.58 | 10.6 | 9.5 | |
| V_2_EV_2_ | MESLLP [(VPGVG)_2_VPGEG(VPGVG)_2_]_10_V | 21562.08 | 21569.67 ± 8.50 | 3.26 | 4.3 | |
| V_2_KV_2_- V_2_EV_2_ | MESLLP[(VPGVG)_2_VPGKG(VPGVG)_2_]_10_[(VPGVG)_2_VPGEG(VPGVG)_2_]_10_V | 42326.78 | 42349.00 ± 2.65 | 5.44 | 8.1 | |
| V | MESLLP(VPGVG)_50_V | 21262.25 | 21261.00 ± 1.73 | 4 | 4.3 | |
| V-V | MESLLP(VPGVG)_100_V | 41736.54 | 41590.33 ± 9.81 | 4 | 4.4 | |
| ^a^The theoretical molecular weight (Theo. Mw ) and isoelectric point (pI) was calculated with the ProtParam tool.^[1]^ ^b^The experimental Mw (Exp. Mw) was measured by mass spectroscopy (Figure S2). | | | | | |  |

**Table S2.** Amino acid composition of the recombinant IDPP library determined by HPLC amino-acid analysis after acid hydrolysis.

For each construct theoretical residue counts (Theo) derived from the designed sequences are compared with experimental counts (Exp) obtained from purified samples. The close agreement between Theo and Exp across all constructs confirms the expected sequences and high sample purity.

|  |  | **V_2_EV_2_** | | **V_2_KV_2_** | | **V_2_KV_2_-** **V_2_EV_2_** | | **V** | | **V-V** | |
| --- | --- | --- | --- | --- | --- | --- | --- | --- | --- | --- | --- |
|  | AA | Theo | Exp | Theo | Exp | Theo | Exp | Theo | Exp | Theo | Exp |
| D | ASP |  |  |  |  |  |  |  |  |  |  |
| E | GLU | 11 | 11.83 | 1 | 1.28 | 11 | 8.56 | 1 | 0.98 | 1 | 2.81 |
| N | ASN |  |  |  |  |  |  |  |  |  |  |
| S | SER | 1 | 0.87 | 1 | 1.26 | 1 | 1.31 | 1 | 1.07 | 1 | 1.41 |
| Q | GLN |  |  |  |  |  |  |  |  |  |  |
| H | HIS |  |  |  |  |  |  |  |  |  |  |
| G | GLY | 100 | 103.94 | 100 | 102.06 | 200 | 199.07 | 100 | 99.32 | 200 | 199.12 |
| T | THR |  |  |  |  |  |  |  |  |  |  |
| R | ARG |  |  |  |  |  |  |  |  |  |  |
| A | ALA |  |  |  |  |  |  |  |  |  |  |
| Y | TYR |  |  |  |  |  |  |  |  |  |  |
| C | CYS |  |  |  |  |  |  |  |  |  |  |
| V | VAL | 91 | 87 | 91 | 89.2 | 181 | 178.36 | 101 | 100.98 | 201 | 199.53 |
| M | MET | 1 | 1.18 | 1 | 1 | 1 | 1.05 | 1 | 1 | 1 | 1.15 |
| W | TRP |  |  |  |  |  |  |  |  |  |  |
| F | PHE |  |  |  |  |  |  |  |  |  |  |
| I | ILE |  |  |  |  |  |  |  |  |  |  |
| L | LEU | 2 | 2.01 | 2 | 2.09 | 2 | 2.75 | 2 | 2.26 | 2 | 2.12 |
| K | LYS |  |  | 10 | 9.67 | 10 | 9.99 |  |  |  |  |
| P | PRO | 51 | 51.31 | 51 | 51.2 | 101 | 106.23 | 51 | 51.09 | 101 | 100.05 |

**Table S3.** pK_a_ values of glutamate residues in V_2_EV_2_ and V_2_KV_2_-V_2_EV_2_ as a function of temperature, determined by acid-base titration. Reported values correspond to the mean ± standard deviation (*N = 3*).

|  |  | Temperature | | | | | | | |
| --- | --- | --- | --- | --- | --- | --- | --- | --- | --- |
|  | 5°C | 10°C | 15°C | 25°C | 40°C | 45°C | 50°C | 55°C | 60°C |
| V_2_EV_2_ | 4.24 ± 0.02 | 4.29 ± 0.05 | 4.30 ± 0.02 | 4.33 ± 0.03 | 4.41 ± 0.10 | 4.51 ± 0.12 | 4.55± 0.14 | - | 4.70 ± 0.14 |
| V_2_KV_2_- V_2_EV_2_ | 4.23 ± 0.15 | 4.24 ± 0.21 | 4.29 ± 0.08 | 4.43 ± 0.09 | 4.50 ± 0.15 | 4.62 ± 0.02 | 4.45 ± 0.07 | 4.31 ± 0.19 | 4.15 ± 0.14 |

**Table S4.** Enthalpy (ΔH°) and entropy (ΔS°) values obtained by Van’t Hoff analysis.

| IDPP | ΔH°  (kcal mol^-1^) | | ΔS°  (cal mol^-1^ K^-1^) | |
| --- | --- | --- | --- | --- |
|  | (T < *T*_t_) | (T > *T*_t_) | (T < *T*_t_) | (T > *T*_t_) |
| V_2_EV_2_ | -1.61 ± 0.35 | -6.69 ±0. 68 | -25.39 ± 1.23 | -41.56 ± 2.11 |
| V_2_KV_2_- V_2_EV_2_ | -3.44 ± 0.44 | 14.85 ± 0.26 | -33.5 ± 1.5 | 9.43 ± 0.80 |

**Figure S1.** **SDS-PAGE electrophoresis of the recombinant IDPP library**.

Lanes 1 and 4 correspond to Pierce™ Unstained Protein MW Marker (ThermoFisher Scientific), with molecular weights indicated on the left. Lanes 2, 3, 5, and 6 show the purified IDPPs. The gel was stained with CuCl_2_. The presence of single, sharp bands confirms the high purity and homogeneity of the expressed proteins.

**Figure S2. MALDI-TOF mass spectra of the IDPPs.**

Spectra correspond to (a) V_2_KV_2_, (b) V_2_EV_2_, (c) V_2_KV_2_- V_2_EV_2_ diblock, (d) V [(VPGVG)50], and (e) V-V diblock [(VPGVG)100]. Minor peaks at approximately half the molecular weight correspond to doubly charged species (m/2z).

**Figure S3. Turbidity profiles of individual V_2_EV_2_ and V_2_KV_2_ IDPPs at pH 7.**

UV/Vis turbidity measurements as a function of temperature at 50 µM in ultrapure water (for V_2_EV_2_ and V_2_KV_2_ alone). No detectable LCST transition was observed under the tested conditions, indicating the absence of phase separation for either charged IDPP individually in water.

**Figure S4. Colocalization of oppositely charged IDPPs within binary condensates.**

Confocal fluorescence images of condensates formed by A647-labeled V_2_EV_2_ + FITC-labeled V_2_KV_2_ in solution after 1 h incubation above *T*_t_. The merged image shows the overlap between both fluorescent signals, indicating co-partitioning of both oppositely charged polymers within the same condensate phase. Individual red and green channels are shown separately for comparison.

**Figure S5. Time evolution of V_2_KV_2_-V_2_EV_2_** **condensates in solution.**

Representative confocal microscopy images showing the temporal evolution of condensates formed by the diblock IDPP V_2_KV_2_-V_2_EV_2_ at 250 µM in type I water. Samples were prepared on glass-bottom chambers treated with Pluronic F-127 to reduce nonspecific surface adsorption. (a) Z-stack images acquired at 25 µm above the glass surface, showing condensate evolution in the bulk solution. (b) Z-stack images acquired 1 µm above the glass surface, showing the near-surface condensate population. (c) Three-dimensional projections reconstructed from confocal z-stacks, illustrating the spatial evolution over time.

**Figure S6. Confocal imaging and size quantification of IDPP condensates after 1 h incubation above *T*_t_.** (a) Representative confocal microscopy image of the condensates formed by V_2_KV_2_-V_2_EV_2_ at 250 µM in type I water and imaged in a chamber with pristine glass, without Pluronic F-127 treatment. Under these conditions, the diblock condensates showed lower interaction with the glass interface, allowing visualization of spherical condensates. (b) Quantification of condensate diameters from confocal images after 1 h incubation above *T*_t_. For V, V-V, and V_2_EV_2_ + V_2_KV_2_, condensates were quantified from samples prepared in Pluronic F-127-treated chambers to minimize nonspecific surface interactions. For V₂KV₂-V₂EV₂, due to its pronounced surface wetting behavior, diameters were quantified from condensates observed on pristine glass *(n = 30*).

**Figure S7. Confocal microscopy monitoring of temperature-induced IDPP condensation after microfluidic encapsulation in microdroplets.**

Representative confocal fluorescence micrographs showing condensate formation after encapsulation of the IDPP solutions in water-in-oil microfluidic droplets and thermal triggering of phase separation. Consistent with the LCST-driven spinodal demixing behavior described for elastin-like polypeptides in confined droplets, phase separation proceeded through the appearance of IDPP-rich domains followed by coalescence and coarsening.^[2]^ The charged binary mixture, A647-labeled V_2_EV_2_ + FITC-labeled V_2_KV_2_, and the covalently linked diblock, FITC-labeled V_2_KV_2_-V_2_EV_2_, evolved toward one dominant rounded condensate within each confined droplet, indicating extensive coalescence of the dense phase. In the binary mixture, the overlap between the FITC and A647 signals indicates recruitment of both oppositely charged components into the same dense phase. By contrast, the hydrophobic IDPPs (TAMRA-labeled V and TAMRA-labelled V-V) retained multiple IDPP-rich domains within individual microdroplets, indicating less complete coarsening within the observation window.

**Figure S8. Representative DSC thermograms measured at 5% (w/v) in PBS.** (a) Thermograms of mixtures of V_2_KV_2_ and V_2_EV_2_ at different molar ratios (from 0:100 to 100:0). (b) Thermograms of the diblock copolymer V_2_KV_2_-V_2_EV_2_ and the non-charged IDPPs V (VPGVG)_50_ and V–V (VPGVG)_100_.

**Figure S9. Secondary structure analysis of the IDPPs.**

Below the transition temperature (T < *T*_t_) (a), all IDPPs exhibit disordered conformations characterized by a strong negative minimum near 195 nm and a weak shoulder around 220 nm. Above the *T*_t_ (b), the spectra change markedly: the minimum at 195 nm decreases, indicating reduced random-coil content, while the shoulder near 220 nm becomes more intense and slightly shifts toward 230 nm depending the composition.

**Figure S10. BeStSel deconvolution of the secondary structures found in the IDPP condensates (above the transition temperature).**

Deconvolution of CD spectra using the BeStSel algorithm revealed that all condensates display mixed conformational content rather than a purely disordered signature. The spectra consistently show ~49–51% “Others” (random coil) and ~13.9–15.0% turns, together with a substantial β-structure contribution of ~32–36%. The β fraction is dominated by antiparallel components (Anti2 + Anti3), increasing from ~26.8% in the physically mixed V_2_KV_2_ + V_2_EV_2_ system to ~34.5% in the covalent V_2_KV_2_-V_2_EV_2_ diblock, ~36.3% in V, and ~34.3% in V-V. Notably, only the V_2_KV_2_ + V_2_EV_2_ mixture exhibits a parallel β component (~5.2%) and a minor helical contribution (~2.5%), both absent in V_2_KV_2_-V_2_EV_2_, and the hydrophobic analogues, V, and V-V (≤0.3% parallel; 0% helix). These results indicate that although the condensates remain predominantly disordered, they contain significant β-structures whose orientation depends on polymer architecture: physical mixing (V_2_EV_2_ + V_2_KV_2_) promotes a modest parallel arrangement and trace helicity, whereas covalent linkage (V_2_KV_2_-V_2_EV_2_) or longer chain length (V-V) favor antiparallel β enrichment.

**Figure S11. Temperature-modulated DSC (TM-DSC) deconvolution of enthalpic components for V_2_EV_2_ and V_2_KV_2_ (5% w/v in water).** Samples were adjusted to pH values below or above the side-chain pK_a_ to suppress electrostatic charges (V_2_EV_2_, pH 3; V_2_KV_2_, pH 12). Non-reversing (slow) and reversing (fast) enthalpic contributions are shown, highlighting the effect of protonation state on cooperative dehydration during LLPS. (*N=3*)

**Figure S12. ITC thermograms for the titration of V_2_EV_2_ into V_2_KV_2_.**

(a-d) Raw heat-flow traces from four independent isothermal titration calorimetry (ITC) experiments in which V_2_EV_2_ was titrated into V_2_KV_2_. Experiments were performed in type I water at 60 °C, above the *T*_t_ of the V_2_EV_2_ + V_2_KV_2_ mixture.

**Figure S13. ITC thermograms for the titration of V_2_KV_2_ into V_2_EV_2_.**

(a-d) Raw heat-flow traces from four independent isothermal titration calorimetry (ITC) experiments in which V_2_KV_2_ was titrated into V_2_EV_2_. Experiments were performed in type I water at 60 °C, above the *T*_t_ of the V_2_EV_2_ + V_2_KV_2_ mixture.

**Figure S14. Atomistic MD analyses supporting temperature-dependent compaction, desolvation, intermolecular association, and asymmetric counterion coordination in V_2_EV_2_ + V_2_KV_2_ mixtures.**

(a) Time evolution of intermolecular hydrogen bonds between V_2_EV_2_ and V_2_KV_2_ chains below and above *T*_t_.
(b) Time evolution of the hydrogen bonds formed by the ionizable side chains of V_2_EV_2_ (Glu) and V_2_KV_2_ (Lys) with water.
(c) Time evolution of the intramolecular hydrogen bonds within V_2_EV_2_ and V_2_KV_2_ chains below and above *T*_t_.
(d) Time evolution of hydrogen bonds formed between each IDPP and solvent below and above *T*_t_.
(e) Radial distribution functions (RDFs) between charged residues and counterions for V_2_EV_2_ and (f) V_2_KV_2_.
(g) Time evolution of the radius of gyration (R_g_) and (f) solvent-accessible surface area (SASA) for both chains.

**Figure S15. NMR characterization of the V_2_EV_2_ system in solution.**

(a) Proton-labeling scheme used for residue assignments. Each proton is annotated as $X_{y}^{(n)}$, where X denotes the amino acid residue, *y* denotes the proton position within the residue ((a–e, corresponding to Cα–Cε)), and *(n)* indicates the peptide monomer (0: MESLLP leader; I: VPGVG; II: VPGEG). Because the spectra were acquired in D_2_O, all exchangeable N–H protons, including backbone amides, undergo rapid H/D exchange and are therefore not detected.
(b) ^1^H–^13^C HSQC spectrum (0.6-2.6 ppm, ^1^H dimension). This region contains -CH_3_ groups from Val and Leu ($V_{c}^{I+II}$, $L_{d}^{0}$), methylene (-CH_2_-)groups (e.g., $V_{b}^{I+II}$, $E_{b,c}^{II}$, $M_{c,d}^{0}$, $P_{b,c}^{I+II}$).
(c) ^1^H–^13^C HSQC spectrum (3.0-4.8 ppm) displaying the region that is dominated by Cα–H correlations from Val, Pro, Glu, Lys, and Ser ($V_{a}^{I,II}$, $P_{a}^{I+II}$, $E_{a}^{II}$, $S_{a}^{0}$). This region also includes the intense Cα–CH₂ signals of Gly ($G_{a}^{I+II}$) and those corresponding to Ser Cβ ($S_{b}^{0}$) and Pro Cδ ($P_{d}^{I+II}$).
(d) ^1^H–^1^H zTOCSY spectrum below the *T*_t_ revealing intraresidue spin-system correlations in agreement with HSQC assignments.

**Figure S16. NMR characterization of the V_2_KV_2_ in solution.**

(a) Proton-labeling scheme used for residue assignments. Each proton is annotated as $X_{y}^{(n)}$, where X denotes the amino acid residue, *y* denotes the proton position within the residue (a–e), and *(n)* indicates the peptide monomer (0: MESLLP leader; I: VPGVG; III: VPGKG).
(b) ^1^H–^13^C HSQC spectrum (0.6-2.6 ppm, ^1^H dimension). This region contains -CH_3_ groups from Val and Leu ($V_{c}^{I+III}$, $L_{d}^{0}$), the characteristic Lys side-chain methylenes ($K_{b,c,d,e}^{III}$), as well as -CH_2_- signals from Met and Pro ($M_{c,d}^{0}$, $P_{b,c}^{I,III}$).methylene (-CH_2_-)groups (e.g., $V_{b}^{I+III}$, $M_{c,d}^{0}$, $P_{b,c}^{I+III}$).
(c) ^1^H–^13^C HSQC spectrum (3.0-4.8 ppm) displaying the region that is dominated by Cα–H correlations from Val, Pro, Glu, Lys, and Ser ($V_{a}^{I,III}$, $P_{a}^{I+III}$, $S_{a}^{0}$). This region also includes the intense Cα–CH₂ signals of Gly ($G_{a}^{I+III}$) and those corresponding to Ser Cβ ($S_{b}^{0}$) and Pro Cδ ($P_{d}^{I+III}$).
(d) ^1^H–^1^H zTOCSY spectrum below the *T*_t_ revealing intraresidue spin-system correlations in agreement with HSQC assignments.

**Figure S17. NMR characterization of the V_2_KV_2_–V_2_EV_2_ system in the soluble state (below the *T*_t_, T = 25 °C).**

(a) Proton-labeling scheme used throughout the NMR analysis. Each signal is annotated as $X_{y}^{(n)}$, where X denotes the amino acid residue, *y* denotes the proton position within the residue (a–e), and *(n)* indicates the peptide monomer (0: MESLLP leader; I: VPGVG; II: VPGEG; III: VPGKG). Only protons directly bound to carbon atoms are labeled.
(b) ^1^H–^13^C HSQC spectrum (0.6-3.0 ppm, ^1^H dimension) showing -CH_3_ groups from Val and Leu ($V_{c}^{I+II+III}$, $L_{d}^{0}$), methylene (-CH_2_-)groups from Lys, Glu, Met, and Pro (e.g., $K_{b\text{–}e}^{III}$, $E_{b,c}^{II}$, $M_{c,d}^{0}$, $P_{b,c}^{I+II+III}$), and additional Cβ resonances from the peptide 0-I.
(c) ^1^H–^13^C HSQC spectrum (3.0-4.8 ppm) displaying the region that is dominated by Cα–H correlations from Val, Pro, Glu, Lys, and Ser ($V_{a}^{I,II,III}$, $P_{a}^{I+II+III}$, $E_{a}^{II}$, $K_{a}^{III}$, $S_{a}^{0}$). This region also includes the intense Cα–CH₂ signals of Gly ($G_{a}^{I+II+III}$) and those corresponding to Ser Cβ ($S_{b}^{0}$) and Pro Cδ ($P_{d}^{I+II+III}$).
(d) ^1^H–^1^H zTOCSY spectrum below the *T*_t_ revealing intraresidue spin-system correlations in agreement with HSQC assignments.

**Figure S18. NMR characterization of the V_2_EV_2_ + V_2_KV_2_ system in the soluble state (below the *T*_t_, T = 25 °C).**

(a) Proton-labeling scheme used throughout the NMR analysis. Each signal is annotated as $X_{y}^{(n)}$, where X denotes the amino acid residue, *y* denotes the proton position within the residue (a–e), and *(n)* indicates the peptide monomer (0: MESLLP leader; I: VPGVG; II: VPGEG; III: VPGKG). Only carbon-bound protons are labeled. Because measurements were performed in D₂O, exchangeable protons (amide NH and the Lys NH₃⁺ protons) are absent due to rapid H/D exchange.
(b) ^1^H–^13^C HSQC spectrum (0.6-3.0 ppm, ^1^H dimension) showing -CH_3_ groups from Val and Leu ($V_{c}^{I+II+III}$, $L_{d}^{0}$), methylene (-CH_2_-)groups from Lys, Glu, Met, and Pro (e.g., $K_{b\text{–}e}^{III}$, $E_{b,c}^{II}$, $M_{c,d}^{0}$, $P_{b,c}^{I+II+III}$), and additional Cβ resonances from the peptide 0-I.
(c) ^1^H–^13^C HSQC spectrum (3.0-4.8 ppm) displaying the region that is dominated by Cα–H correlations from Val, Pro, Glu, Lys, and Ser ($V_{a}^{I,II,III}$, $P_{a}^{I+II+III}$, $E_{a}^{II}$, $K_{a}^{III}$, $S_{a}^{0}$). This region also includes the intense Cα–CH₂ signals of Gly ($G_{a}^{I+II+III}$) and those corresponding to Ser Cβ ($S_{b}^{0}$) and Pro Cδ ($P_{d}^{I+II+III}$).
(d) ^1^H–^1^H zTOCSY spectrum below the *T*_t_ revealing intraresidue spin-system correlations in agreement with HSQC assignments.

**Figure S19. NMR characterization of the V polymer in the soluble state (below the *T*_t_, T = 25 °C).**

(a) Proton-labeling scheme used throughout the NMR analysis. Each signal is annotated as $X_{y}^{(n)}$, where X denotes the amino acid residue, *y* denotes the proton position within the residue (a–e), and *(n)* indicates the peptide monomer (0: MESLLP leader; I: VPGVG). Only protons directly bound to carbon atoms are labeled.
(b) ^1^H–^13^C HSQC spectrum (0.6-2.6 ppm, ^1^H dimension) showing -CH_3_ groups from Val and Leu ($V_{c}^{I}$, $L_{d}^{0}$), the β-methylene (Cβ) of Val ($V_{b}$), and the Cγ and Cδ methylenes of Pro ($P_{b,c}$), in addition to the methylene (-CH_2_-)groups from the leader peptide 0.
(c) ^1^H–^13^C HSQC spectrum (3.0-4.8 ppm) displaying the region that is dominated by Cα–H correlations from Val, Pro, Glu, Lys, and Ser ($V_{a}^{I}$, $P_{a}^{I}$, $S_{a}^{0}$). This region also includes the intense Cα–CH₂ signals of Gly ($G_{a}^{I}$) and the only two -CH_2_- resonances appearing above 3 ppm, corresponding to Ser Cβ (S$S_{b}^{0}$) and Pro Cδ ($P_{d}^{I}$).
(d) ^1^H–^1^H zTOCSY spectrum below the *T*_t_ revealing intraresidue spin-system correlations in agreement with HSQC assignments.

**Figure S20. ^1^H–^15^N HMBC characterization of the V_2_E V_2_ + V_2_K V_2_ system.**

(a) Proton-nitrogen labeling scheme used for ^15^N assignments. Each nitrogen correlation is annotated using blue uppercase letters (A–F), corresponding to distinct ^15^N chemical environments arising from N-terminus, Lys primary amine, and backbone amide positions within the two-component (V_2_E V_2_ + V_2_K V_2_) system.

(b) 2D ^1^H-^15^N HMBC spectra acquired below the *T*_t_ (25 °C), where the mixture remains fully solvated.

(c) Magnified view (zoom-in) of panel (b), highlighting the nitrogen region between –230 and –280 ppm. This zoom resolves overlapping correlations and improves visualization of nitrogen signals A, B, C, D and F.

(d) ¹H–¹⁵N HMBC spectrum above the *T*_t_ (60 °C), where V_2_E V_2_ + V_2_K V_2_ system is condensed. (e) Zoom-in of panel (d), resolving the remaining nitrogen signals in the –230 to –280 ppm region. Only nitrogen A–D remained observable. Cross-peaks corresponding to Glu amide nitrogen (F) and the Lys ε-amine (E) were no longer detected.

The loss of signals E and F is attributed both to natural-abundance limitations of ^15^N, together with severe line broadening due to rapid transverse relaxation (short T_2_) associated with the restricted molecular motion and increased microviscosity in the dense phase. These effects suppress long-range ^1^H-^15^N correlations for dynamically sensitive nitrogen environments.

**Figure S21. 2D ^1^H-^1^H NOESY spectra of charged IDPPs measured below (a) and above (b) the transition temperature (*T*_t_).**

2D NOESY spectra of the physical mixture V_2_EV_2_ + V_2_KV_2_ and the covalently bonded V_2_KV_2_- V_2_EV_2_ acquired at 25 °C (T < *T*_t_) and 60 °C (T > *T*_t_) in D_2_O to monitor temperature-induced changes in proton-proton correlations. Despite the clear temperature-dependent changes observed in the NOESY spectra, no unambiguous cross-peak indicative of Glu-Lys side-chain association could be identified in the condensed phase. This negative result likely arises from two factors. First, the β-methylene protons of Glu and the ε-methylene protons of Lys might be separated by distances that exceed the ∼5 Å spatial limit required for observable NOE build-up, making direct through-space detection intrinsically unlikely even if salt-bridge formation occurs. Second, above the *T*_t_ the spectra undergo severe line broadening due to reduced molecular mobility in the condensed phase, which further suppresses NOE intensities and leads to overlap with nearby aliphatic resonances.

**Figure S22. 2D ^1^H-^1^H NOESY spectra of hydrophobic IDPPs measured below (a) and above (b) the transition temperature (*T*_t_).**

Spectra of V and V-V IDPPs acquired at 25 °C (T < *T*_t_) and 60 °C (T > *T*_t_) under identical conditions to the charged systems. Below the *T*_t_, both samples display sharp, well-resolved cross-peaks characteristic of flexible, hydrated chains in the soluble state. Above the *T*_t_, the spectra exhibit a pronounced broadening of cross-peaks, more evident for the V-V, consistent with reduced molecular mobility within the condensate. This broadening reflects increased chain entanglement and slower tumbling associated with phase condensation, rather than specific side-chain interactions.

**Figure S23. Representative ^1^H-NMR spectra of charged IDPP condensates (expanded region, 2.5–4.0 ppm) recorded at different times after surpassing the transition temperature (T > *T*_t_).**

Spectra of (a) the physical mixture V_2_EV_2_ + V_2_KV_2_ and (b) the covalently bonded V_2_KV_2_-V_2_EV_2_ were collected at 60 °C at selected time points (0, 1, 2, 8, 43, and 200 min). The expanded region focuses on the ε-methylene protons of lysine, which serve as sensitive reporters of local microenvironment and ionization state. Over time, a progressive broadening and attenuation of the signals is observed, consistent with aggregation and reduced molecular mobility within the condensed phase. Around 3.2-3.1 ppm, two partially resolved populations appear: one corresponding to free Lys–NH₃⁺ groups and the other to Lys-NH_2_-H-Glu salt-bridge formation, revealing the gradual formation of electrostatic interactions during condensate maturation.

**Figure S24. Multiphase organization of V_2_KV_2_-V_2_EV_2_ + V condensates on untreated glass.**

Confocal fluorescence micrograph of multicomponent condensates formed by FITC-labeled V_2_KV_2_-V_2_EV_2_ diblock (green) and TAMRA-labeled V (blue) at 250 μM in type I water after 1 h of incubation in a chamber with untreated glass. V segregated into internal compartments within the V_2_KV_2_-V_2_EV_2_-rich condensates, reproducing the multiphase organization observed on Pluronic-F127 treated glass. These results indicate that internal demixing is not driven by Pluronic-mediated surface wetting or substrate interactions, but reflects an intrinsic difference in miscibility between the diblock condensate and non-charged analogues. Scale bar: 20 μm.

**References**

[1]	J. M. Walker, E. Gasteiger, C. Hoogland, A. Gattiker, S. Duvaud, M. R. Wilkins, R. D. Appel, A. Bairoch, *The Proteomics Protocols Handbook* **2005**, 571.

[2]	J. R. Simon, N. J. Carroll, M. Rubinstein, A. Chilkoti, G. P. López, *Nat. Chem.* **2017**, *9*, 509.
